# Differential Affinity and Catalytic Activity of CheZ in *E. coli* Chemotaxis

**DOI:** 10.1371/journal.pcbi.1000378

**Published:** 2009-05-08

**Authors:** Siebe B. van Albada, Pieter Rein ten Wolde

**Affiliations:** FOM Institute for Atomic and Molecular Physics, Amsterdam, The Netherlands; University of Illinois at Urbana-Champaign, United States of America

## Abstract

Push–pull networks, in which two antagonistic enzymes control the activity of a messenger protein, are ubiquitous in signal transduction pathways. A classical example is the chemotaxis system of the bacterium *Escherichia coli*, in which the kinase CheA and the phosphatase CheZ regulate the phosphorylation level of the messenger protein CheY. Recent experiments suggest that both the kinase and the phosphatase are localized at the receptor cluster, and Vaknin and Berg recently demonstrated that the spatial distribution of the phosphatase can markedly affect the dose–response curves. We argue, using mathematical modeling, that the canonical model of the chemotaxis network cannot explain the experimental observations of Vaknin and Berg. We present a new model, in which a small fraction of the phosphatase is localized at the receptor cluster, while the remainder freely diffuses in the cytoplasm; moreover, the phosphatase at the cluster has a higher binding affinity for the messenger protein and a higher catalytic activity than the phosphatase in the cytoplasm. This model is consistent with a large body of experimental data and can explain many of the experimental observations of Vaknin and Berg. More generally, the combination of differential affinity and catalytic activity provides a generic mechanism for amplifying signals that could be exploited in other two-component signaling systems. If this model is correct, then a number of recent modeling studies, which aim to explain the chemotactic gain in terms of the activity of the receptor cluster, should be reconsidered.

## Author Summary

In both prokaryotes and eukaryotes, extra- and intracellular signals are often processed by biochemical networks in which two enzymes together control the activity of a messenger protein via opposite modification reactions. A well-known example is the chemotaxis network of *Escherichia coli* that controls the swimming behavior of the bacterium in response to chemical stimuli. Recent experiments suggest that the two counteracting enzymes in this network are colocalized at the receptor cluster, while experiments by Vaknin and Berg indicate that the spatial distribution of the enzymes by itself can markedly affect the response of the network. We argue using mathematical modeling that the most widely used model of the chemotaxis network is inconsistent with these experimental observations. We then present an alternative model in which part of one enzyme is colocalized with the other enzyme at the receptor cluster, while the remainder freely diffuses in the cytoplasm; moreover, the fraction at the cluster both binds more strongly to the messenger protein and modifies it faster. This model is consistent with a large number of experimental observations and provides a generic mechanism for amplifying signals.

## Introduction

The protein network that controls chemotaxis of *Escherichia coli* is arguably the most-studied and best-characterized signal transduction pathway. Its relative simplicity makes it an ideal model system for studying signal amplification, integration, transduction, and adaptation. The network consists of three parts: i) a cluster of receptors at the cell membrane, which detects the extracellular ligand; ii) the intracellular signaling pathway, which transmits the signal from the receptor cluster to the flagellar motors; iii) the network that controls the response of the flagellar motors. The intracellular signaling pathway is a push-pull network that consists of a kinase, CheA, that phosphorylates the messenger protein CheY and a phosphatase, CheZ, that dephosphorylates the phosphorylated messenger protein CheY_p_. In wild-type cells, CheA is localized exclusively at the receptor cluster, and also CheZ is predominantly localized at the receptor cluster [1]. Recently, however, Vaknin and Berg studied mutants in which CheZ can no longer bind the receptor cluster, as a result of which it is uniformly distributed in the cytoplasm [2]. They observed that the response of the intracellular signaling pathway of these mutant cells differs strongly from that of wild-type cells. Inspired by this observation, we recently performed a mathematical modeling study of a canonical push-pull network, which showed that the spatial distribution of the antagonistic enzymes by itself can have a dramatic effect on the response [3]. Our study also showed, however, that the effect depends upon the regime in which the network operates. Here, we first address by detailed mathematical analysis of the canonical model of the *E. coli* chemotaxis network whether the difference in response between wild-type and CheZ mutant cells can be explained by the different spatial distribution of CheZ in these cells. We find that this is not the case; also realistic changes in parameters such as rate constants and protein concentrations do not seem sufficient to explain the difference in response. We then consider two refinements to the canonical model. First, we study the effect of cooperative dephosphorylation of CheY_p_ by CheZ [4]–[7]. Next, we consider a refined model of the intracellular chemotaxis network of *E. coli*, in which a small fraction of CheZ is localized at the receptor cluster, while the remainder is distributed in the cytoplasm. This model, which is supported by a wealth of experimental data, can explain many of the experimental observations of Vaknin and Berg [2], and it provides a novel mechanism for signal amplification.

The canonical model of the intracellular chemotaxis network of *E. coli* is described by the following set of chemical reactions:
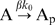
(1)

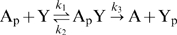
(2)

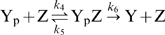
(3)


In this network, the phosphorylated form of the messenger, CheY_p_ (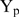), transmits the signal from the receptor cluster to the flagellar motors. The phosphorylation level of CheY is regulated by a kinase CheA (A) and a phosphatase CheZ (Z). CheY_p_ also exhibits autophosphorylation and autodephosphorylation, but these reactions are much slower than phosphorylation by CheA and dephosphorylation by CheZ, respectively. The input to the signal transduction pathway is 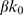, where 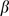 is a parameter between zero and one that reflects the activity of the receptor cluster and 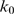 denotes the maximum rate of autophosphorylation of CheA. The value of 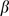 depends on the ligand concentration [L]: 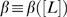; 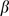 shifts to lower (higher) values upon the addition of attractant (repellent). In order for *E. coli* to adapt to a changing ligand concentration, the activity of the receptor cluster, 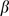, is also modulated by the methylation and demethylation enzymes CheR and CheB, respectively.

In wild-type *E. coli* cells, not only CheA, but also CheZ is localized at the receptor cluster [1]. In these cells, CheZ is anchored to the receptor cluster by CheA [8],[9]. In a recent experiment, Vaknin and Berg compared the response of wild-type cells to that of CheZ mutant cells, in which CheZ does not bind to CheA, but diffuses in the cytoplasm [2]. They studied the response of the chemotaxis network by measuring the interaction between CheZ and CheY_p_ using FRET imaging. While the input of the network was thus the concentration of ligand, the measured output was proportional to the total, integrated concentration of CheY_p_ bound to CheZ, 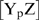 (see also Eq. 3).

Vaknin and Berg found that the colocalization of the antagonistic enzymes has a marked effect on the dose-response curve [2]. In wild-type cells, in which CheA and CheZ are colocalized at the receptor cluster, the response of 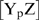 to changes in the concentration of the attractant serine is more sensitive than in mutant cells, in which CheZ is distributed in the cytoplasm. Moreover, in *cheRcheB* cells, which lack the methylation and demethylation enzymes, the response to the addition of serine is also sharper when CheA and CheZ are colocalized at the receptor cluster [2].

In the next section, we show that the experiments of Vaknin and Berg [2] impose strong constraints on any model that aims to describe the intracellular chemotaxis network. In the subsequent section, we argue that the canonical model does not meet these constraints: neither changes in the spatial distribution of CheZ, nor realistic changes in the rate constants and protein concentrations seem sufficient to explain the differences in the response curves of the mutant and wild-type cells. Indeed, we argue that the experiments of Vaknin and Berg demonstrate that the canonical model needs to be augmented.

In the subsequent sections, we present two refined models of the intracellular chemotaxis network of *E. coli*, which both can explain the difference in response between wild-type cells and CheZ mutant cells, as measured by Vaknin and Berg [2]. The first model assumes that 1) in wild-type cells, CheZ is localized at the cluster, while in the CheZ mutant cells, CheZ freely diffuses in the cytoplasm; 2) CheZ in wild-type cells has a higher phosphatase activity than CheZ in the CheZ mutant cells, as suggested by the observation of Wang and Matsumura that interactions of CheZ with CheA enhance its phosphatase activity [10]; 3) CheZ in wild-type cells acts non-cooperatively, while CheZ in the mutant cells acts cooperatively, as motivated by the experimental observations of [4],[6],[7]. While this model can describe the FRET response curves as measured by Vaknin and Berg [2], it assumes that in wild-type cells *all CheZ proteins* are bound at the cluster. However, the experiments of Vaknin and Berg show that in wild-type cells, only a small fraction of CheZ is bound at the receptor cluster; the remainder freely diffuses in the cytoplasm [2].

In the next section, we therefore present an alternative model. The key ingredients of this model are: 1) in wild-type cells, a small, yet significant, fraction of CheZ is bound to the receptor cluster, while the remainder freely diffuses in the cytoplasm [2]; 2) the fraction of CheZ at the cluster has a higher binding affinity for the substrate CheY than that of cytosolic CheZ; 3) the catalytic activity of CheZ bound to the cluster is higher than that of CheZ in the cytoplasm. This model bears similarities to that recently proposed by Lipkow [11], although our model neither requires oligomerization of CheZ at the receptor cluster nor shuttling of CheZ between the cytoplasm and the receptor cluster. In the section *Differential affinity and catalytic activity* we show using a simplified model how the combination of differential binding affinity and differential catalytic activity provides a novel mechanism for amplifying signals: As the activity of the receptor cluster and hence that of the kinase CheA is increased from zero and CheY becomes phosphorylated, CheY_p_ first binds CheZ at the receptor cluster; only when CheZ at the receptor cluster is saturated, does CheY_p_ bind CheZ in the cytoplasm; since CheZ at the cluster has a higher catalytic activity than CheZ in the cytoplasm, the response of CheY_p_ is sigmoidal. Finally, we also incorporate cooperative binding of CheY_p_ to CheZ [5]–[7] into the model and show that this model can explain the response of *E. coli* to changes in serine concentration, as measured by Vaknin and Berg [2].

## Results

### Decomposing the response

Vaknin and Berg performed experiments on four bacterial strains: wild-type cells, *cheRcheB* cells lacking the methylation and demethylation enzymes CheR and CheB, CheZ mutant cells, and CheZ mutant cells lacking CheR and CheB [2]. Analysis of their dose-response curves 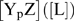 (the concentration of CheY_p_CheZ—a CheY_p_ molecule bound to a CheZ dimer—as a function of the ligand concentration L) is complicated by the fact that they are determined by both the response of the receptor cluster, 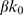, to the change in the ligand concentration, [L], and by the response of the intra-cellular signaling pathway, 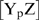, to changes in the activity of the receptor cluster, 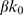. However, these two networks can be viewed as two independent modules connected in series, which can be analyzed separately, as we discuss below. Moreover, this modularity means that the dose-response curves, 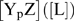, of the four strains can be obtained by multiplying the response curves of the two modules.

The first module is the receptor cluster. Its activity, 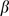, depends upon the concentration of ligand, [L], and upon the methylation states of the receptors, which is controlled by the methylation and demethylation enzymes CheR and CheB, respectively. However, the dynamics of receptor methylation and demethylation by CheR and CheB are much slower than that of receptor-ligand (un)binding and phosphorylation and dephosphorylation of CheY; in fact, this separation of time scales allows *E. coli* to both respond and adapt to a changing ligand concentration. This separation of time scales also makes it possible to model the response to ligand at short time scales without explicitly taking into account the (de)methylation dynamics; the absence of CheR and CheB in 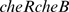 cells, will lead to different methylation states of the receptors, yet can be modeled implicitly by taking different functional forms for 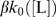. For wild-type cells, the response of the cluster is thus characterized by the response function 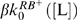, while for *cheRcheB* cells, the response is described by 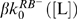.

The second module of the chemotaxis network, the intracellular signal transduction pathway, is described by the set of reactions in Equations 1–3. The input of this network is 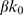, while the output is the concentration of CheY_p_, 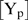, or, as in the experiments of Vaknin and Berg, the total concentration of CheY_p_ bound to CheZ, 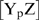 [2]. The response curve of this network, 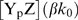, depends upon the nature of CheZ, and will thus be different for wild-type cells and CheZ mutant cells. Importantly, 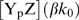 is independent of the methylation states of the receptors. We assume that 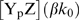 also does not depend upon the presence of CheB, although phosphorylated CheA can phosphorylate not only CheY but also CheB, leading to another form of adaptation on a time scale longer than that of the response; we will come back to this in the *Discussion* section. Thus, we assume that 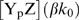 of 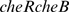 cells is the same as that of wild-type cells; the absence of CheR and CheB in *cheRcheB* cells only affects 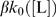. Hence, the response of the intra-cellular signaling pathway in wild-type cells is characterized by the response function 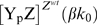, while the response of CheZ mutant cells is characterized by 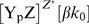.

**Figure 1:**
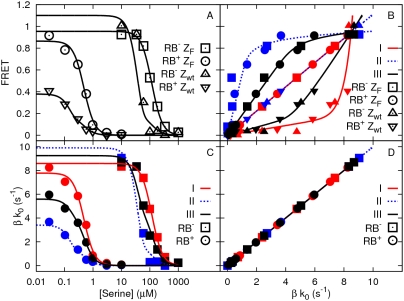
Three models that reproduce the response curves of Ref. [2]. A. The four response curves of Figure 5a in [2], rescaled according to Figure 1 of Text S1 and assuming a total concentration 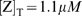. Model I (red data) is based on a linear dependence 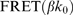 for cells containing the non-localizing phosphatase mutant CheZ (see panel B). As a consequence, the activity of the receptor cluster in panel C is proportional to the FRET signal for CheZ mutant cells in panel A. The response 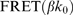 for cells containing wild-type CheZ is extremely sharp for model I (see panel B). Model II (blue data) is based on a linear function of 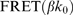 for cells with wild-type CheZ. As a consequence, 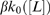 is proportional to the dose-responses curve for cells with wild-type CheZ (compare panels A and C). In this case, the response curve 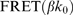 for CheZ mutant cells is very concave. Model III was constructed by assuming that 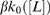 is a linear combination of the response functions of models I and II. The resulting response functions 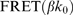 in panel B are less extreme than those of models I and II. The straight line 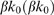 in panel D helps to visualize the projection between panels B and C.

If the receptor cluster and the intracellular chemotaxis pathway indeed behave as two independent modules connected in series, then the response function 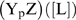 should be given by the composite function 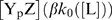. Hence, the response function of the four strains in Ref. [2] should be of the form: 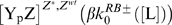. As we show in Figure 1 of Text S1, the experiments of Vaknin and Berg on the four different strains provide strong evidence for the hypothesis that the receptor cluster and the intracellular network are indeed two independent modules connected in series. Yet, these experiments do not uniquely prescribe how the overall response is decomposed. This is illustrated in Figure 1, which show the response curves of three different models, indicated by different colors, that all can explain the dose-response curves of Figure 1A. Each model consists of the functions 
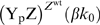
 and 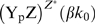 (Figure 1B), corresponding to wild-type and CheZ mutant cells respectively, and the functions 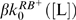 and 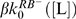 (Figure 1C), corresponding to cells containing CheR and CheB and *cheRcheB* cells lacking CheR and CheB, respectively. For each model, the four composite functions 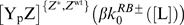 exactly reproduce the four dose-response curves of Figure 1A. Model I (red lines and points) relies on the assumption that 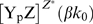 is a straight line over the concentration range of interest (see Figure 1B). This means that 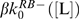 and 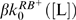 are proportional to 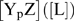 of CheZ mutant cells lacking CheR and CheB and CheZ mutant cells containing CheR and CheB, respectively; this can be verified by comparing Figure 1A to Figure 1C. The experiments of Vaknin and Berg [2] now fully determine the function 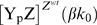, which can be constructed from 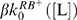 and 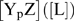 of the wild-type cells, and 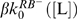 and 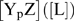 of the *cheRcheB* cells (see Figure 1B); this function has a strongly convex shape. Model II (blue lines and points) relies on the assumption that 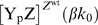 is a linear function (see Figure 1B). In this case 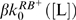 and 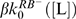 are proportional to 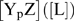 of wild-type and *cheRcheB* cells, respectively (see Figure 1A and Figure 1C). The functional form of 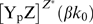 of CheZ mutant cells now has a concave shape (see Figure 1B). These two models are two extreme scenarios that both can explain the data shown in Figure 1A.

In the following sections we will also consider models that have less extreme functional forms for 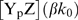; these models lie in between model I and model II. We construct such models, starting from models I and II, by defining functions 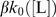 as linear combinations 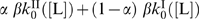, where 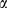 is a parameter between zero and one; for 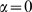 the model reduces to model I, while for 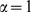 the model reduces to model II. Model III (black lines and points) was constructed by putting 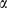 equal to 0.5. For this model, 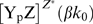 of CheZ mutant cells is slightly concave, whereas 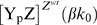 of wild-type cells is slightly convex.

The model that can describe the response of 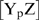 to changes in ligand concentration should not only be able to reproduce the dose-response curves of Figure 1, it should also satisfy other important conditions. Most importantly, wild-type cells can chemotax, which means that in their non-stimulated state they can respond to the addition as well as to the removal of attractant. Bacteria lacking 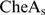 are able to chemotax towards attractants as well, although less efficiently than wild-type bacteria [12]. These mutants are probably similar to CheZ mutants in that the binding of CheZ to the receptor cluster is hampered in both strains. The requirement that both strains can chemotax means that the concentration of CheY_p_ in the non-stimulated state should be within the working range of the motor, i.e. between 1 and 5 µM [13],[14].

### Original model: The canonical push-pull network

We now address the question whether the canonical model for the chemotaxis pathway of *E. coli*, as given by Equations 1–3, can describe the experimental results of Vaknin and Berg [2]. We first study the effect of the spatial distribution of CheZ, thus leaving the other parameters unchanged. As we will show, the spatial distribution of CheZ alone is not sufficient to explain their experimental results. We will then also vary rate constants and concentrations to see whether the canonical model can describe these results.

To elucidate the effect of CheZ localization, we have computed the input-output relations for a network in which CheA and CheZ are colocalized at the receptor cluster (corresponding to wild-type cells) and for a network in which CheA is localized at the receptor cluster, while CheZ is distributed in the cytoplasm (corresponding to CheZ mutant cells); for both networks, the chemical reactions are given by Equations 1–3. The steady-state input-output relations of these networks were obtained numerically by discretizing the system on a 1D grid and propagating the chemical rate equations, which are given in the *Methods* section, in space and time until steady state was reached.

As pointed out in the previous section, the input of the intracellular network is not directly the ligand concentration [L], but rather 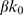 (see Eq. 1), which implicitly depends upon [L]. Importantly, we first assume that the functional dependence of 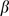 on the ligand concentration [L], as well as the rate constants of all the reactions, is the same for wild-type and CheZ mutant cells: this allows us to elucidate the effect of colocalization of the antagonistic enzymes on the input-output relations. The model and the values of its parameters were taken from Sourjik and Berg [14].

**Figure 2:**
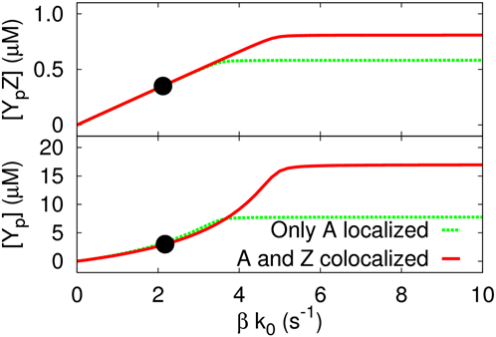
Total, integrated concentration of CheY_p_ bound to CheZ, 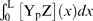, and CheY_p_, 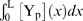, as a function of 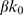 for the canonical model of the chemotaxis network of *E. coli*, shown in Equations 1–3. The red curves correspond to wild-type cells in which CheA and CheZ are colocalized at the receptor cluster, while the green curves correspond to the mutant cells in which CheA is localized at the pole, while CheZ freely diffuses in the cytoplasm. The bullets correspond to the non-stimulated state of the system. The diffusion constant of the diffusing components is 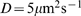 [38]. For other parameter values, see [14].

The principal results of our calculations are shown in Figure 2. This figure shows for wild-type and CheZ mutant cells, the concentration of CheY_p_CheZ (a CheY_p_ molecule bound to a CheZ dimer) and the concentration of CheY_p_ as a function of 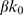 (see Equation 1); the bullets correspond to the non-stimulated state of the network [14]. Figure 2 shows that the model predicts that the spatial distribution of CheZ affects the response to the addition of repellent or the removal of attractant, which corresponds to an increase in 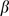. More importantly, the model predicts that the CheZ distribution should not affect the response to the addition of attractant: When 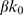 is lowered from its value 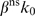 in the non-stimulated state, both the change in 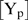 and 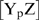 do not depend much on the spatial distribution of CheZ. This result is thus in contrast with the drastic effect of enzyme localization on the response found by Vaknin and Berg [2].

The network given by Equations 1–3 is very similar to a canonical push-pull network, in which two enzymes covalently modify a substrate in an antagonistic manner [15] (see Text S2 for how these networks can be mapped onto each other). We have recently studied in detail the effect of enzyme localization on the response of a push-pull network [3]. Our principal finding is that enzyme localization *can* have a marked effect on the gain and sensitivity of push-pull networks, seemingly consistent with the experiments of Vaknin and Berg [2], but contradicting the numerical results shown in Figure 2. The resolution of this paradox is that both the quantitative and qualitative consequences of enzyme localization depend upon the regime in which the push-pull network operates. In particular, if the activation rate is independent of the substrate concentration and if the deactivation rate is linear in the messenger concentration, then phosphatase localization has no effect on the response curve [3]. This is precisely the case for the chemotaxis network studied here. For 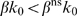, CheZ is unsaturated [14] and the dephosphorylation rate of CheY_p_ is thus proportional to 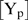. The influx 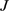 of CheY_p_ is constant, i.e. independent of [Y]. This is not because the phosphorylation reaction is in the zero-order regime; this reaction is, in fact, in the linear regime [14]. The influx 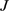 of CheY_p_ at the cell pole is constant because a) in steady state 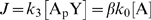 and b) in the weak activation regime CheA is predominantly unphosphorylated (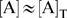), which means that 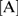 is fairly insensitive to the spatial distribution of CheZ. Hence, according to the model of Equations 1–3, in this regime the concentration of CheY_p_ does not depend upon the spatial distribution of CheZ, which is indeed what Figure 2 shows.

However, while the model of Equations 1–3 predicts that in wild-type cells the response of [Y_p_Z] to the addition of attractant does not depend on the location of CheZ, the experiments by Vaknin and Berg clearly demonstrate that it does [2]. What could be the origin of the discrepancy between the model predictions and the experimental results of Vaknin and Berg? As mentioned above, the response of [Y_p_Z] to the ligand concentration [L] depends upon the response of [Y_p_] to the activity of the receptor cluster, 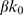, and upon the response of 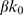 to the ligand concentration [L]. If we keep with the assumption that the functional dependence of 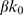 on [L], *βk*_0_([L]), is the same for both wild type and CheZ mutant cells, the discrepancy between the predictions of the canonical model and the experimental observations of Vaknin and Berg must lie in the dependence of [Y_p_Z] on 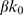. It is quite likely that the rate constants and/or concentrations that are used in the calculations differ from those *in vivo*. It is also possible that the topology of the canonical model of the intracellular chemotactic pathway, Eqs. 1–3, is incorrect. In order to discriminate between these two scenarios, we will, in the rest of this section, first address the question whether it is possible to explain the experimental observations with the canonical model by allowing for different values of parameters such as rate constants and protein concentrations. We will then argue that simply allowing for different parameter values is probably not sufficient to explain the experiments of Vaknin and Berg, and that thus the canonical model should be reconsidered.

Irrespective of the model parameters, it is always true that the rate of phosphorylation equals the rate of dephosphorylation if the system is in steady state. For the canonical model, i.e. Equations 1–3, this means that for both the spatially uniform network in which CheA and CheZ are colocalized, and the spatially non-uniform network in which CheZ is distributed in the cytoplasm, the following relation holds in steady state:
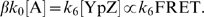
(4)Here, “FRET” denotes the FRET signal, which is proportional to the total, integrated, concentration of CheY_p_ bound to CheZ, [Y_p_Z]. For the regime of interest, 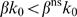, the concentration of unphosphorylated CheA, [A], is essentially constant for the conventional model, because only a small fraction of the total amount of CheA is phosphorylated; below we discuss scenarios in which this relation might not hold. Equation 4 thus shows that if 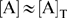, the FRET signal only depends upon the activity of the receptor cluster, 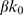, and upon the phosphatase activity, 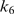, but not upon other rate constants in the network, nor upon the expression levels of, for instance, CheY and CheZ. Moreover, if 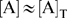, the FRET signal, in this model, is *linear* in the activity of the receptor cluster: 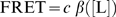, where 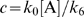 is the proportionality constant. Incidentally, this explains the linear dependence of [Y_p_Z] on 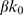 for 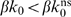 in Figure 2B.

The linear relation between [YpZ] and 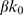 as predicted by the canonical model would mean that the dose-response curves, i.e. FRET([L]), solely reflect the response of the receptor cluster to the addition of ligand, *βk*_0_([L]). Vaknin and Berg report the *renormalized* FRET response: they normalize the FRET signal at ligand concentration [L] to the FRET signal at zero ligand concentration, 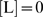 [2]. If the response of [Y_p_Z] to 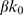 would indeed be linear, then the renormalized FRET signal would be given by 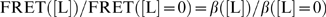. Hence, the proportionality factor 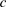 would drop out. The renormalized FRET signal would thus be given by the dependence of the activity of the receptor cluster on the ligand concentration, *βk*_0_([L]). While plotting the renormalized FRET signal may mask potentially useful information, this observation does allow us to draw an important conclusion: *If βk*_0_([L]) *is the same for wild type and CheZ mutant cells, and as long as* 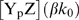 *is linear, the canonical model cannot describe the experiments of Vaknin and Berg, even if we allow for different parameter values for the rate constants or protein concentrations*.

The experiments of Wang and Matsumura illustrate the importance of this conclusion [10]. Their experiments suggest that the phosphatase activity is enhanced by its interaction with CheA_s_, which is localized at the receptor cluster [10]. This would predict that in the CheZ mutant cells (in which CheZ is distributed in the cytoplasm), the phosphatase activity is lower. This could either be due to a decrease in the CheZ-CheY_p_ association rate 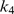, or to a decrease in the catalytic activity 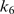. Eq. 4 reveals that a change in the association rate 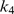 has no effect on the FRET response curve, as long as 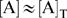. In contrast, a change in 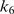 would change the dependence of [Y_p_Z] on 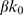 (see Equation 4); in particular, decreasing 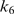 would increase the slope. However, as long as the dependence of [Y_p_Z] on 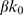 is linear, the renormalized FRET response would still be given by *βk*_0_([L]): merely changing the slope of [Y_p_Z] as a function 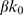 does not change the renormalized FRET response. More in general, only allowing for different rate constants or protein concentrations between the wild-type cells and mutant cells is not sufficient to explain the data, if indeed *βk*_0_([L]) is the same for both cells and 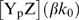 is linear.

The critical ingredient in the above analysis is that [Y_p_Z] varies linearly with 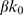, both for the wild-type and the CheZ mutant cells. We now first address the question whether deviations from this linear relation could explain the data, and then how these deviations might arise. The simplicity of the canonical model, Equations 1–3, does not allow for a convex dependence of [Y_p_Z] on 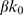. Figure 1B then immediately shows that any model that aims to describe the dose-response curves of both the wild-type cells and the CheZ mutant cells, should exhibit a linear relationship 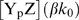 for wild-type cells and a concave function 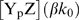 for CheZ mutant cells (blue data set). To generate a non-linear response of [Y_p_Z] as a function of 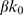 over the concentration range of interest, the condition 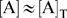, which was the critical condition to generate a linear relationship (see Equation 4), should be violated; this means that 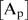 should increase significantly within the concentration range of interest. An inspection of the canonical network, Equations 1–3, reveals that 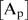 increases more rapidly with 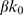, when 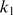, 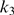, 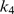 or 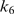 decrease (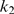 and 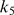 are very small, and can thus be neglected). The effect of changing these parameters can be understood by considering the following relations in steady state: 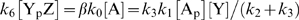. For example, as 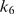 decreases, 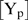 and [Y_p_Z] tend to increase, and 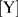 tends to decrease; the latter means that to obey the above relations, 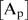 should increase. Decreasing 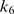 thus means that [Y_p_Z] as a function of 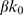 not only has a higher initial slope, but also levels off more rapidly because 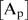 increases: the function becomes concave for lower values of 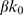. Similarly, it can be deduced that while a decrease of 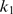 does not change the initial slope of 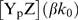 (because for low 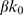, 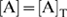, and the slope is then independent of 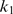 (see Equation 4)), it does lower the value of 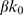 at which 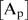 increases; again the function becomes concave for lower values of 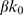.

Changes in the rate constants (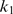, 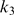, 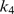, 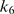) could thus potentially explain the dose-response curves measured by Vaknin and Berg [2]. We have tested by extensive numerical calculations, in which we did not only change these rate constants but also protein concentrations, whether changing these parameters can indeed explain the experiments. The results are shown in Text S2. The calculations reveal that changing 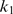 and 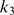 does not have a large effect (see Figures 4 and 5 of Text S2); moreover, it does not seem likely that changing CheZ affects the binding of CheY to CheA, although this cannot be ruled out. Changing 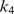 and 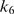 has a stronger effect: assuming that 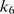 in the CheZ mutant cells is a factor 10 lower than 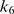 in the wild-type cells yields a reasonable fit to the FRET data of Vaknin and Berg [2] (see Figure 7 of Text S2).

Do the CheZ mutant cells exhibit a tenfold lower phosphatase activity (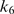)? The canonical model with the assumption that in the CheZ mutant cells the phosphatase activity is ten times lower is an example of model I discussed in the previous section (blue lines in Figure 1B). While this model could explain the FRET data of Vaknin and Berg, it should be realized that according to this model the CheZ mutant cells would be tumbling all the time: as Figure 7 of Text S2 shows, in the non-stimulated state the concentration of CheY_p_ would be at its maximal value, and the clockwise bias would be close to unity. However, the experiments of Sanatinia *et al.* [12] show that both the wild-type and the mutant bacteria can chemotax, which suggests that not only in the wild-type cells, but also in the CheZ mutant cells, 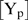 is within the working range of the motor when the cells are in their non-stimulated state. We therefore present two new models. In the next section, we consider a model of type I, in which the FRET signal in wild type cells is proportional to the activity of the receptor cluster 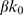, whereas the response curve 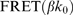 for mutant cells is strongly concave. In the subsequent section, we consider a model of type III that exhibits a weakly concave response curve 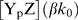 for the CheZ mutant cells, and, consequently, a convex response curve 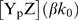 for the wild-type cells.

### The cooperative model

Recent experiments strongly suggest that the intracellular chemotaxis network of *E. coli* has a more complicated topology than that of the canonical push-pull network discussed in the previous section. In particular, in the canonical model discussed above the phosphatase reactions were described by simple Michaelis-Menten reactions. However, experiments of Eisenbach and coworkers [4],[6] and Silversmith *et al.* [7] have shown that the activity of CheZ depends in a cooperative manner on the CheY_p_ concentration. It is clearly important to understand how the response curve 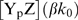 is affected by the cooperative dependence of phosphatase activity on CheY_p_ concentration. In this section, we present a simple model for the cooperative dependence of the phosphatase activity on CheY_p_ concentration, which can be solved analytically. Furthermore, we show that incorporation of cooperativity into the phosphatase reactions can lead to a model of type I (see Figure 1) and therefore gives a possible explanation for the experiments by Vaknin and Berg [2].

In *vitro* data [4],[6],[7] suggest that the activity of CheZ depends in a cooperative manner on the CheY_p_ concentration. The experiments of Eisenbach and coworkers [4],[6] suggest that the activity of CheZ also depends in a cooperative manner on the CheZ concentration, suggesting that CheZ may oligomerize upon CheY_p_ binding [4]–[6]. Other biochemical *in vitro* experiments [16] and more recent *in vivo* FRET experiments [9], however, do not provide support for this idea. We therefore assume that the activity of CheZ in the mutant cells only depends cooperatively on the CheY_p_ concentration.

The model for the cooperative dephosphorylation of CheY_p_ by CheZ is based upon the following assumptions: 1) a single CheZ dimer can bind up to two CheY_p_ molecules; 2) CheZ can dephosphorylate CheY_p_ in both CheY_p_-bound states, thus dephosphorylation can occur when only a single CheY_p_ molecule is bound or when two CheY_p_ molecules are bound. This model can be described by two coupled Michaelis-Menten reactions, those of Eq. 3 in combination with
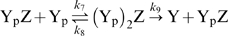
(5)In steady state, the phosphatase activity is given by
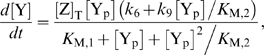
(6)where 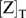 is the total concentration of CheZ and 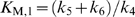 and 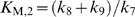 are the Michaelis-Menten constants of Equation 3 and Equation 5, respectively (see Text S3 for a derivation). It can be seen that if 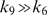 and if 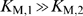, the dephosphorylation rate is given by
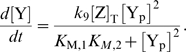
(7)This is a Hill function with a Hill coefficient of 2 and a concentration at which the rate is half maximal (the inflection point) given by 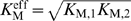. Clearly, strong cooperativity arises when 3) the binding of the first substrate molecule facilitates the binding of the second one, making 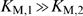 and 4) the catalytic activity is higher when two substrate molecules are bound than when one is bound, i.e. 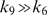. In Text S3 we give an extended analysis of this model, which shows that it can fit the *in vitro* data of Blat and Eisenbach [6] not only qualitatively, but also quantitatively; this fit satisfies criteria 3) and 4). Recently, Silversmith *et al.* independently developed a similar model as that of Eqs. 3 and 5 on the basis of their *in vitro* experiments [7], although they did not present the analytical result of Eq. 6 [7]. Interestingly, their model also satisfies criterion 3): binding of the first CheY_p_ molecule facilitates the binding of the second CheY_p_ molecule. However, in their model binding of the second CheY_p_ molecule does not enhance the catalytic activity of CheZ [7], in contrast to our model. We cannot obtain a good fit to the *in vitro* data of Eisenbach and coworkers [6], nor, as discussed below, to the *in vivo* data of Vaknin and Berg [2], without relaxing criterion 4). Finally, we would like to emphasize that the rate constants derived from fitting *in vivo* data may differ from those obtained from fitting *in vitro* data. In particular, diffusion-limited reaction rates will often be lower in living cells due to a lower diffusion constant, and a detailed analysis of this model (see Text S3) suggests that in this system this might be the case.

**Figure 3:**
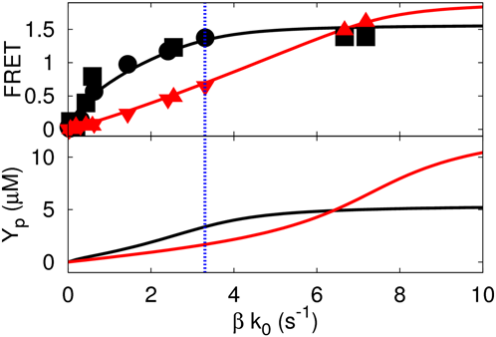
FRET vs. 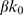 and [Y_p_] vs. 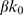 for the best fit of the cooperative model (Equations 1–3 and 5). In this model, with 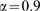 (see Figure 1), it is assumed that in wild-type cells all CheZ proteins are bound to the receptor cluster, while in the CheZ mutant cells all CheZ proteins diffusive in the cytoplasm. The chemical reactions of this model are given by Eqs. 1–3 and Eq. 5, for both cells. The black line and symbols correspond to CheZ mutant cells, while the red line and symbols correspond to wild type cells. The dotted vertical line denotes the value of 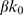 in the non-stimulated state. The FRET signal is assumed to be proportional to 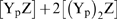. The smaller concentration of CheY_p_ in mutant cells when 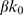 is large is due to the fact that in these cells, CheZ diffuses (see also Ref. [3]). The values of the rate constants that are the same for wild-type and CheZ mutant cells are: 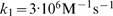, 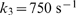, 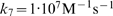, 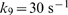; the values of the rate constants that are different between wild-type and CheZ mutant cells are: 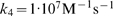 for wild type cells and 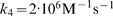 for mutant cells, 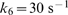 for wild type cells and 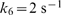 for mutant cells. The total concentrations are 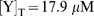, 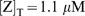 and 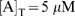 (for parameter values, see [6],[14],[39]). The diffusion coefficient of all cytosolic components is set to 5 µm^2^ s^−1^; all enzyme-substrate dissociation rates are zero.

In the model presented in this section, we assume that in wild-type cells all CheZ proteins are localized at the receptor cluster, while in the CheZ mutant cells all CheZ proteins freely diffusive in the cytoplasm. For both cells, the chemical reactions are given by Eqs. 1–3 and Eq. 5. However, while the rate constants of the phosphorylation reactions in Eqs. 1 and 2 are identical for both cells, they differ for the dephosphorylation reactions of Eqs. 3 and 5. In particular, in order to obtain a good fit to the FRET data [2], we have to assume that in the CheZ mutant cells CheZ acts cooperatively, while in the wild-type cells CheZ acts non-cooperatively. Specifically, while for the wild-type cells, not only the two CheY_p_-CheZ association rates 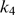 and 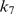, but also the two catalytic activities 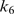 and 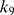 can be assumed to be identical—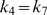; 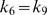
—, for the CheZ mutant cells it is required that 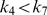 and 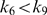 (see caption of Figure 3 for parameter values).

The results for this model are shown in Figure 3. The FRET response of wild-type cells is similar to that in the canonical model discussed in the previous section; it is essentially linear in 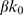 over the relevant range of 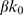, because CheZ acts non-cooperatively. However, the FRET response of wild-type cells is weaker than that of CheZ mutant cells over this range. This is because the catalytic activity of CheZ with one CheY_p_ molecule bound, 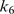, is higher in wild-type cells than in CheZ mutant cells. Indeed, this model would suggest that the interaction of CheA with CheZ enhances the catalytic activity of CheZ when one CheY_p_ molecule is bound to CheZ. Another important point to note is that the FRET response of CheZ mutant cells is strongly concave over the relevant range of 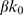. This model is indeed an example of type I, as discussed in the section *Decomposing the response*. The concave FRET response of CheZ mutant cells is a consequence of the cooperative dephosphorylation of CheY_p_ by CheZ: for small receptor activities 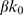, [Y_p_] is low, CheZ is mostly singly occupied by CheY_p_, and since the catalytic activity of CheY_p_CheZ, 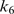, is relatively small (as compared to that of 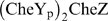, 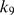), a given increase in 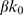 must be balanced by a relatively large increase in 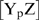 and hence the FRET signal; for higher 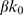, 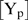 increases, CheZ becomes doubly occupied with CheY_p_, and since 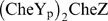 has a higher catalytic activity than CheY_p_CheZ, a given increase in receptor activity 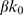 is balanced by a relatively small increase in 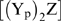. Indeed, if 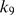 would be similar to 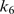, as Silversmith *et al.* propose [7], the FRET response of the CheZ mutant cells would not be concave, and no good fit to the data of Vaknin and Berg [2] could be obtained.

### Differential affinity and catalytic activity of CheZ

While the model discussed in the previous section can describe the FRET response as measured by Vaknin and Berg [2], it also assumes that in wild-type cells all CheZ proteins are localized at the receptor cluster. However, the data of Vaknin and Berg [2] suggest that only a small fraction of CheZ is localized at the receptor cluster. We therefore present here an alternative model, which, in our opinion, is consistent with the currently available experimental data.

#### The Model

The key ingredients of our model are:

1. *In wild-type cells, a small fraction of CheZ, of 10–20%, is bound to the receptor cluster, while the remainder diffuses freely through the cytoplasm.* Figure 2b of Vaknin and Berg [2] shows the cyan signal, coming from CFP fused to CheZ, *after the addition of attractant*. This signal represents the spatial distribution of CheZ. The figure suggests that about 10–20% of CheZ is bound to the receptor cluster, with the remainder more or less homogeneously distributed in the cytoplasm. This estimate is consistent with that based on the known chemistry of CheZ binding to the receptor cluster. CheZ can be localized to the receptor cluster via binding to CheA, which is part of the receptor cluster. CheA exists in two forms, CheA_s_ and CheA_L_, which can form the following dimers: CheA_L_CheA_L_, CheA_L_CheA_s_, and CheA_L_CheA_s_. The first two, CheA_L_CheA_L_ and CheA_L_CheA_s_, have catalytic activity and can transfer phosphoryl groups to CheY [17]–[20]; the third, the homodimer CheA_s_CheA_s_, does not have catalytic activity, but can bind CheZ. Earlier experiments suggest that CheZ binds selectively to CheA_s_ [8],[10],[21], although recent FRET experiments indicate that CheZ also binds to CheA_L_ [9]. Following Lipkow [11], we estimate that the number of CheA_s_CheA_s_ homodimers is about 360, while the number of CheZ dimers is about 1600 [22]. If we assume that CheZ predominantly binds CheA_s_CheA_s_, and that each of the CheA_s_CheA_s_ homodimers strongly binds one CheZ dimer, we arrive at the estimate that about 20% of the CheZ dimers is bound to the cluster, consistent with the estimate based on the FRET data of Vaknin and Berg [2].
2. *In wild-type cells, CheY_p_ has a much higher affinity for CheZ bound to CheA than for CheZ freely diffusing in the cytoplasm.* Figure 3a of Vaknin and Berg [2] shows that in non-stimulated cells containing wild-type CheZ, the total amount of [Y_p_Z] in the cytoplasm roughly equals that of [Y_p_Z] at the receptor cluster; yet, as mentioned above, Figure 2b of Ref. [2] shows that the total amount of CheZ at the cluster is about 10–20% of that in the cytoplasm; this means that CheZ bound to CheA at the receptor cluster has a higher affinity for CheY_p_ than CheZ in the cytoplasm, as can also be seen directly from Figure 2d of Ref. [2]. The higher affinity could be due to a lower enzyme-substrate dissociation rate, or a higher enzyme-substrate association rate. We assume that binding of CheZ to CheA increases the association rate. It is conceivable that CheA enhances the CheZ- CheY_p_ association rate in a manner analogous to the gain of function mutations in CheZ studied by Silversmith *et al.* [7]: CheA might relieve inhibition of the binding of CheY_p_ to CheZ. A more speculative hypothesis is that CheA increases the CheZ- CheY_p_ association rate because of the close physical proximity between CheA, where CheY is phosphorylated, and cluster-bound CheZ: a CheY molecule that has just been phosphorylated by a CheA_p_ dimer at the cluster, can very rapidly bind cluster-bound CheZ; in fact, if CheY_p_ would be directly transferred from CheA_p_ to CheZ, the association rate could even exceed the diffusion-limited rate.
3. *In wild-type cells, CheZ bound to CheA at the receptor cluster has a higher phosphatase activity than CheZ in the cytoplasm.* The experiments of Wang and Matsumura [10] suggest that the interaction of CheZ with CheA enhances its dephosphorylating activity. This could either be due to a higher CheZ-CheY_p_ association rate, or to a higher catalytic activity. We assume that binding of CheZ to CheA not only increases the CheZ-CheY_p_ association rate, as discussed above, but also the catalytic activity of CheZ.
4. *In CheZ mutant cells, CheZ cannot bind to CheA at the cluster. CheZ in these cells has the same phosphatase activity and the same binding affinity for CheY_p_ as CheZ in wild-type cells that is not bound to CheA at the cluster.* As crystallographic data [23] and mutagenesis data [20] suggest, we assume that in the CheZ mutant protein only the domain that allows it to interact with CheA is affected; the part that allows the CheZ mutant protein to interact with CheY_p_ is thus assumed to be unaffected. This assumption is not critical for obtaining a good fit of our model to the data of Vaknin and Berg [2]. It is merely a simplifying assumption to reduce the number of free parameters. Indeed, it would be of interest to characterize the enzymatic activity of CheZ F98S—the CheZ mutant used by Vaknin and Berg [2]—since experiments by Silversmith *et al.* show that mutations far from the active site can, in fact, significantly change the enzymatic activity of CheZ [7].

For reasons of clarity, we first disregard the cooperativity in the phosphatase activity of CheZ. The CheZ mutant cells are thus described by the reactions of Eqs. 1–3, while the wild-type cells are described by the reactions of Eqs. 1–2, Eq. 3 for the reactions involving diffusive CheZ and the following reactions involving localized CheZ:
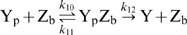
(8)Here, the total concentration of localized CheZ, 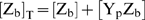, is low as compared to the total concentration of CheZ, 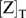. Furthermore, the association rate 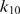 and the catalytic activity 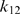 of localized CheZ, are high as compared to the corresponding rates 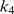 and 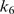 for diffusive CheZ. As we will show below, the critical parameters of this model are the fraction of CheZ bound to CheA at the receptor cluster, the ratio of the association rates 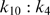 and the ratio of the catalytic activities 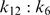.

The model presented here is similar to that of Lipkow [11] in that both assume that part of CheZ can bind the cluster. However, the models also differ in two important aspects: 1) in the model of Lipkow [11], the binding of CheZ to CheA is conditional on the binding of CheZ to CheY_p_; consequently, while in our model the bound fraction of CheZ is fairly constant in time, in the model of Lipkow [11] the amount of CheZ bound to the cluster depends upon the current stimulus level: for instance, in her model, after the removal of attractant, CheZ moves from the cytoplasm to the cluster upon binding of CheY_p_; 2) in the model of Lipkow [11], the binding of one CheY_p_CheZ pair to a CheA homodimer, can nucleate the formation of oligomers of CheY_p_CheZ pairs at the cluster. However, as mentioned above, recent *in vitro* [7],[16] and *in vivo* experiments [9] seem to disprove the idea of CheZ oligomerization. Our calculations reveal that CheZ oligomerization is not necessary; the conditions listed above, are sufficient to explain the FRET data of Vaknin and Berg [2]. Moreover, the relative simplicity of our model makes it possible to elucidate the mechanism by which differential enzyme-substrate binding affinity and differential catalytic activity can sharpen the response curve.

**Figure 4:**
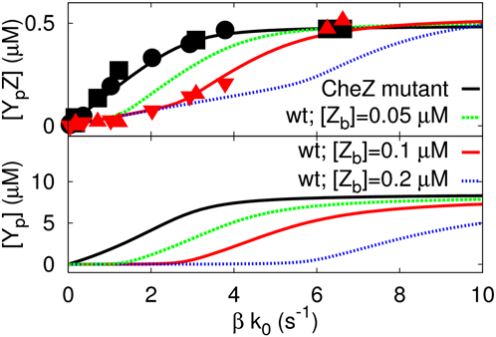
The effect of the total amount of CheZ that is bound to the cluster, as given by 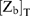, on the response of [Y_p_Z] and [Y_p_] in the differential-affinity-and-catalytic-activity model (Equations 1–3 and Equation 8). The black line corresponds to the prediction of our model for CheZ mutant cells, while the red line corresponds to the model prediction for wild-type cells, in which 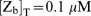. The green and blue dashed lines correspond to the model prediction for wild-type cells with 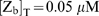 and 0.2 µM, respectively. The symbols correspond to the experimental data of Vaknin and Berg [2]. The circles correspond to CheZ mutant cells with CheR and CheB, the squares correspond to CheZ mutant cells without CheR and CheB, the triangles correspond to wild-type cells and the inverted triangles correspond to wild-type cells without CheR and CheB. Please note that as 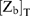 is increased, the inflection point that separates the first from the second regime shifts to higher values of 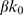 and to higher values of [Y_p_Z]—to a good approximation, at this point 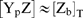. It is also seen that CheY_p_ in the first regime is essentially zero. This is because the phosphatase activity of CheZ at the receptor cluster is much higher than that of CheZ in the cytoplasm. The baseline parameters are: 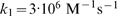, 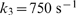, 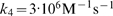, 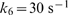, 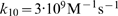, 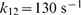, 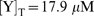, 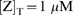, 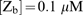 and 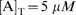 (for parameter values, see [14],[39]). The diffusion coefficient of all cytosolic components is 5 µm^2^ s^−1^; all enzyme-substrate dissociation rates were set to zero.

**Figure 5 pcbi-1000378-g005:**
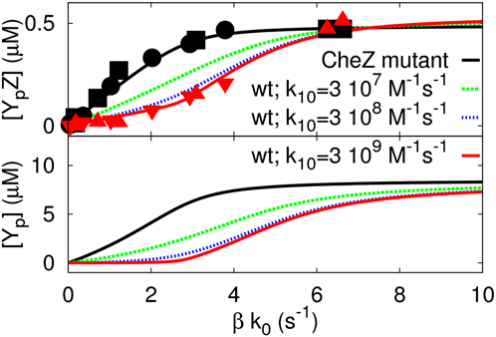
The effect of the rate of association between CheY_p_ and CheZ bound to the receptor cluster, 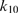, on the response of [Y_p_Z] and [Y_p_] in the differential-affinity-and-catalytic-activity model (Equations 1–3 and Equation 8). The black line and black symbols corresponds to the CheZ mutant cells (see also Figure 4), while the red line and red symbols correspond to cells containing wild-type CheZ, in which 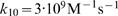; the dashed green and blue lines correspond to CheZ-wild-type cells with 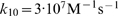 and 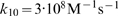, respectively. Please note that as 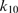 is lowered, the distinction between the two regimes becomes less sharp, because more CheY_p_ molecules diffuse into the cytoplasm before they will bind CheZ molecules. For parameter values, see the caption of Figure 4.

**Figure 6:**
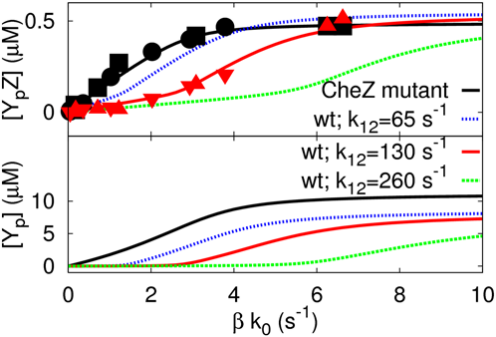
The effect of the catalytic rate of CheZ bound to the receptor cluster, 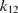, on the response of [Y_p_Z] and [Y_p_] in the differential-affinity-and-catalytic-activity model (Equations 1–3 and Equation 8). The black line and black symbols corresponds to the CheZ mutant cells (see also Figure 4), while the red line and red symbols correspond to CheZ-wild-type cells, in which 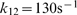; the dashed green and blue lines correspond to CheZ-wild-type cells with 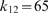 and 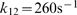, respectively. Please note that as 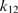 is increased, the initial slope of 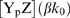 of wild-type cells, which is inversely proportional to 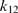, is decreased; the slope of the second regime is, to a good approximation, inversely proportional to the catalytic activity of freely diffusive CheZ, 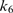, and thus fairly constant. Please also note that since the height of the inflection point is given by 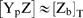 and thus independent of 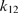, the inflection point shifts to higher values of 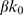 with increasing 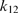.

Figures 4–6 show how the total amount of CheY_p_CheZ pairs and CheY_p_ is affected by varying the critical parameters in this model: the fraction of CheZ bound to the cluster (Figure 4), the rate 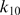 at which CheY_p_ associates with CheZ at the cluster (Figure 5), and the catalytic rate 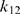 of CheZ at the cluster (Figure 6); the baseline parameters are given in Figure 4. In all figures, the black line corresponds to CheZ mutant cells; the red line corresponds to CheZ wild-type cells with the baseline parameter set; the green and blue lines correspond to the results of the CheZ wild-type cells, where the parameter of interest is either increased or decreased (see caption for parameter values). The black and red symbols correspond to the experimental results of Vaknin and Berg [2], as described in section *Decomposing the response*; the value of 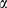 was, somewhat arbitrarily, taken to be 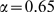, which means that 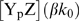 is sigmoidal for CheZ wild-type cells and hyperbolic for CheZ mutant cells. The origin of the hyperbolic curve of the CheZ mutant cells is similar to that which underlies the response curves of the canonical model: 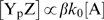, where initially, as 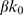 increases from zero, 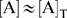 is constant but then decreases as 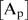 increases significantly (see section *Original Model*). We will now discuss the origin of the sigmoidal curves of 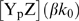 of the wild-type cells.

Figures 4–6 show that the response curves of 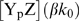 of wild-type cells effectively consist of two parts, corresponding to the binding of CheY_p_ to cluster-bound CheZ and freely diffusive CheZ, respectively. When 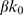 is low, a CheY molecule that has just been phosphorylated by a CheA dimer at the cluster, will most likely bind a CheZ dimer that is bound to the cluster because of the higher association rate between CheY_p_ and cluster-bound CheZ, as compared to that between CheY_p_ and freely diffusive CheZ: 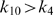. Since cluster-bound CheZ has a high phosphatase activity, the concentration of CheY_p_ and hence CheY_p_ bound to CheZ will initially increase only slowly with 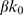. Nevertheless, at some point CheZ at the cluster will become saturated with CheY_p_. At this point 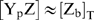. When 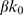 is then increased further, a phosphorylated CheY molecule can no longer bind a cluster-bound CheZ dimer. It will then diffuse into the cytoplasm, where it can bind freely diffusive CheZ. Since the catalytic activity of CheZ in the cytoplasm is lower than that of CheZ bound to CheA at the cluster, [Y_p_Z] and 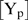 will now quickly rise. This combination of differential affinity and differential catalytic activity thus provides a generic mechanism for enhancing the sharpness of the response.

We can now understand the effect of varying the critical parameters in this model. As the fraction of CheZ that is bound to the cluster increases (from green to red to blue in Figure 4), the amount of CheY_p_ needed to saturate cluster-bound CheZ increases, leading to a shift of the inflection point in 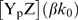 to higher values of 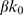. However, while increasing the fraction of cluster-bound CheZ shifts the inflection point to higher values of 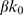, it does not significantly change the initial slope of 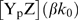, nor does it change the slope 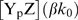 after the inflection point: these slopes are determined by the catalytic activities of cluster-bound CheZ and freely diffusive CheZ, 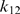 and 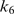, respectively. This can be seen in Figure 6: as the catalytic activity of 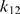 is increased (from blue to red to green), the initial slope of 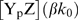 decreases. Please also note that since the slope of 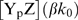 after the inflection point is determined by parameters of freely diffusive CheZ, it is similar to the initial slope of 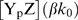 of the CheZ mutant cells, which indeed only contain freely diffusive CheZ, exhibiting the same phosphatase activity as diffusive CheZ in wild-type cells. Figure 5 illustrates the importance of the association rate. As the rate of association between CheY_p_ and cluster-bound CheZ decreases (from red to blue to green), the response curve 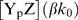 of CheZ cells moves towards that of the CheZ mutant cells. The reason is that as the rate of association between CheY_p_ and cluster-bound CheZ is lowered, it becomes more likely that a phosphorylated CheY molecule diffuses into the cytoplasm, where it will be dephosphorylated by freely diffusive CheZ with a lower catalytic activity.

The differential-affinity-and-activity model is able to explain the measured difference between the response curves for the CheZ mutant cells and the CheZ wild-type cells. However, while the response curves of Vaknin and Berg [2] can be reproduced by the model, this is not the only constraint. As discussed above, both wild-type and CheZ mutant cells should be able to chemotax [12]. This means that the model should give CheY_p_ concentrations between 1 and 5 µM for both strains in the non-stimulated state [14]. As can be seen from the fit used in Figures 4–6, in the CheZ mutant, the CheY_p_ concentration is 8 µM in the non-stimulated state, which is well outside this range.

This fit can, however, be improved by taking into account the effect of cooperativity in the phosphatase reactions, which we have neglected thus far in the differential-affinity-and-activity model. The reactions of diffusive CheZ, both in the wild-type cells and in the CheZ mutants cells, are given by Eqs. 3 and 5, while the reactions involving CheZ localized at the receptor cluster in wild-type cells are given by Eq. 8 in combination with
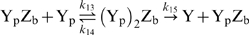
(9)As before, we assume that both the affinity to CheY_p_ and the phosphatase activity of CheZ are enhanced when CheZ is localized to CheA at the receptor cluster. This means that the association rates 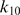 and 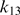 are much larger than the corresponding association rates for cytosolic CheZ, and that the catalytic activity 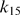 is larger than the catalytic activity 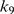 for cytosolic CheZ.

**Figure 7:**
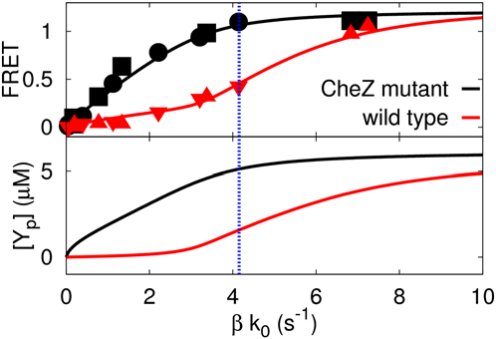
FRET vs. 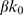 and [Y_p_] vs. 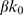 for the best fit of the full differential-affinity-and-catalytic-activity model, which includes cooperativity in CheZ (Equations 1–3, and Equations 5, 8 and 9). The black line and symbols correspond to CheZ mutant cells, while the red line and symbols correspond to cells containing wild-type CheZ (see also Figure 4). The FRET signal is assumed to be proportional to 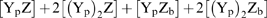; the value of 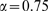 (see Figure 1). The dotted vertical line denotes the value of 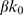 in the non-stimulated state. The parameter values are 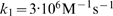, 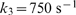, 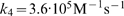, 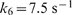, 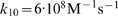, 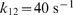, 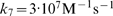, 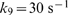, 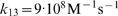, 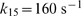; 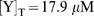, 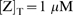, 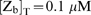 and 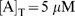 (for parameter values, see [6],[14],[39]). The diffusion coefficient of all cytosolic components is set to 5 µm^2^ s^−1^; all enzyme-substrate dissociation rates are zero.

Figure 7 shows 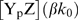 and 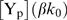 for CheZ wild-type cells and CheZ mutant cells [2]. In combination with a response curve for 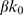 vs. [Serine] with 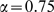, the four dose-response curves in Figures 5a and 5c of Ref. [2] are reproduced. Comparing Figure 7 with Figures 4–6 of the simplified differential-affinity-and-activity model shows that the cooperative dependence of the phosphatase activity on CheY_p_ concentration does not dramatically affect the dose-response curves, a conclusion that was also reached by Sourjik and Berg [14]. Indeed, in this model it is possible to obtain a good fit to the data [2] while assuming that the catalytic activity of CheZ is independent of the number of bound CheY_p_ molecules, as suggested by the *in vitro* observations of Silversmith *et al.* [7] (data not shown); the critical ingredients of this model are that the binding affinity and catalytic activity of cluster-bound CheZ are higher than those of freely diffusive CheZ. As for the model without CheZ cooperativity, 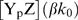 is in agreement with experiment, both for CheZ wild-type and CheZ mutant cells. Moreover, the 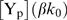 response curve of the CheZ wild-type cells agrees with experiment in the sense that the concentration of CheY_p_ equals 2 µM in the non-stimulated state, which is within the working range of the motor. The concentration of CheY_p_ in the CheZ mutant cells in their non-stimulated state is around 5 µM, which is lower than that in the simplified differential-affinity-and-activity model, but still at the high end of the working range of the motor.

## Discussion

### A new model for the intracellular signaling network

The experiments by Vaknin and Berg on the effect of CheZ localization on the dose-response curves of *E. coli* [2] impose strong constraints on the design of a model of the intracellular chemotaxis network. These experiments unambiguously demonstrate that the second derivative of 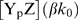 of CheZ wild-type cells is larger than that of CheZ mutant cells (see Figure 1). The topology of the intracellular chemotaxis network of the canonical model (Equations 1–3) is such that the second derivative of 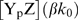 must be equal to or smaller than zero: according to the canonical model the response curve cannot be convex. One way to fit the data is to assume that the response curve 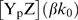 of CheZ wild-type cells is a straight line over the concentration range of interest, while 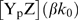 of CheZ mutant cells is concave. The canonical model can yield such response curves. However, this scenario requires that in the CheZ mutant cells, some of the rate constants, such as the phosphatase activity, differ strongly from those in wild-type cells. Moreover, this would mean that CheZ mutant cells would adapt to a state in which 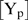 is outside the working range of the motor. This scenario thus seems unlikely, although it cannot be ruled out.

Here, we have presented two different models that can explain the FRET data of Vaknin and Berg [2]. In the first model, 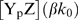 of CheZ wild-type cells is linear, while 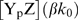 of CheZ mutant cells is strongly concave. The model is based on the *in vitro* observation that CheZ dephosphorylates CheY_p_ in a cooperative manner [5]–[7]. The model leads over the relevant range of interest to fairly similar response curves 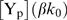 for wild-type and mutant cells, and the non-stimulated state lies around 3 µM. This model, however, assumes that in wild-type cells all CheZ proteins are localized at the receptor cluster, while the data of Vaknin and Berg [2] suggest that in these cells only a fraction of about 10–20% is localized at the receptor cluster.

We have therefore presented an alternative model that is consistent with most, if not all, of the currently available data. In this model, 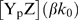 of CheZ wild-type cells is sigmoidal, while 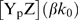 of CheZ mutant cells is hyperbolic. The model relies on the assumption that a small fraction of CheZ is localized at the receptor cluster, while the remainder freely diffuses in the cytoplasm; moreover, it assumes that CheZ localized at the receptor cluster has both a higher binding affinity for CheY_p_ and a higher catalytic activity than CheZ in the cytoplasm. All these assumptions seem to be supported by experiment [2],[10].

In essence, the model that we propose consists of a push-pull network with one activating enzyme, CheA, and two deactivating enzymes, CheZ bound to the cluster and CheZ that freely diffuses in the cytoplasm. Our analysis shows that the competition between these two deactivating enzymes for binding and deactivating the substrate can yield an ultrasensitive response even when the push-pull network does not operate in the zero-order regime. In fact, this mechanism of differential-affinity-and-catalytic-activity is evocative of the “branch point effect”, in which the interdependence of the activities of two branch-point enzymes that compete for a common substrate can yield an abrupt change in the flux through one of the enzymes [24]. In the model proposed here, the spatial dependence of both the substrate-binding affinity and catalytic activity of CheZ only acts to create two types of deactivating enzymes; the proposed scheme could also work in a well-stirred model if one assumes that there exist two deactivating-enzyme species.

### Does the intracellular signaling pathway contribute to the gain?

If the response function 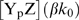 of wild-type cells is sigmoidal, as the differential-affinity-and-catalytic-activity model predicts, then the large number of recent studies on signal amplification by the receptor cluster has to be reconsidered [25]–[33]. If the relation between [Y_p_Z] and 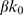 would be linear, as predicted for wild-type cells in the canonical and cooperative model, then the renormalized FRET response would be given by the dependence of the activity of the receptor cluster, 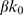, on the ligand concentration [L]. This would justify the studies that describe the ‘front end’ amplification of the chemotaxis network, namely the response of [Y_p_Z] to changes in [L], in terms of the signal amplification properties of the receptor cluster [25]–[33]. However, if the dependence of [Y_p_Z] on the activity of the receptor cluster, 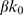, would not be linear, then the front end amplification would not be fully determined by the response of the receptor cluster to changes in the ligand concentration. Indeed, to explain the front-end gain, the extent to which the signal is amplified as it is transmitted from the receptor cluster to CheY_p_CheZ would then also have to be taken into account.

Recently, Kim *et al.* experimentally addressed the question whether CheZ contributes to the gain of the chemotaxis network [34]. To this end, they compared the motor response of wild-type cells to that of 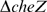 mutant cells in which the activity of the receptor cluster was adjusted by mutating the Tsr receptor to compensate for the change in CheY_p_ levels [34]. They observed that the change in the motor bias upon a change in ligand concentration was similar for these cells, and concluded that CheZ does not contribute to the gain. However, it should be noted that the mutations in the Tsr receptor as made by Kim *et al.* [34] may affect the signal amplification by the receptor cluster, especially since it is believed that interactions between receptors (and even between receptors of different types) strongly affect the gain [25]–[33]. If this would be the case, then the observation that in the “bias adjusted” 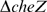 mutant cells the motor response is similar to that of wild-type cells, would imply that CheZ does contribute to the gain. Our analysis supports a scenario in which CheZ contributes to the gain, but cannot rule out the alternative scenario. If CheZ does not contribute to the gain, then 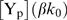 should be the same for wild-type cells and CheZ mutant cells over the relevant range of the activity of the receptor cluster. In our differential-affinity-and-catalytic-activity model, which is consistent with most of the experimental data, the response curves are different (Figure 7), but in our cooperative model they are, in fact, fairly similar (Figure 3). The problem is that while the data of Vaknin and Berg [2] put strong constraints on any model that aims to describe the response of the intracellular signaling pathway, they do not uniquely prescribe it (Figure 1). To elucidate the response of the intracellular signaling pathway and to discriminate between the models that we propose, we believe that FRET measurements should be made of CheY_p_-CheZ and CheY_p_-FLiM interactions [9], not only for wild-type cells, but also for 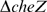 mutants [34] and the CheZ F98S mutants studied by Vaknin and Berg [2].

### The concentration of CheY_p_ in non-stimulated cells

While the differential-affinity-and-catalytic-activity model can describe the dose-response curves as reported by Vaknin and Berg [2], a number of issues remain. The first is that in the full differential-affinity-and-catalytic-activity model, which takes into account CheZ cooperativity, the *total* concentration of [Y_p_] in non-stimulated CheZ mutant cells is on the border of the working range of the motor, while experiments on mutant cells lacking CheA_s_, which plays a role in localizing CheZ to the receptor cluster [12], suggest that CheZ mutant cells can chemotax. This raises an interesting question, which to our knowledge has not been studied yet: How strongly does the efficiency of chemotaxis depend upon the concentration of CheY_p_ in the adapted state? In particular, how well must that be inside the working range of the motor? It is conceivable that cells with [Y_p_] at the high end of the motor's working range can chemotax, albeit less efficiently. Another possibility is that CheZ mutant cells can chemotax, because [Y_p_] forms spatial gradients inside CheZ mutant cells [2]: while [Y_p_] at some motors will be outside the motor's working range, [Y_p_] at other motors might be inside the working range of the motor.

But perhaps the most likely explanation is that phosphorylation of CheB by CheA_p_ provides a negative feedback loop on the activity of the receptor cluster that tends to keep the concentration of CheY_p_ within a certain range. The concentration of CheY_p_ in the adapted state is determined by the activity of the receptor cluster in the adapted state, which is controlled by the activity of the methylation and demethylation enzymes CheR and CheB, respectively. CheA_p_ cannot only phosphorylate CheY, but also CheB. Moreover, phosphorylated CheB has a higher demethylation activity than unphosphorylated CheB. Since CheY and CheB compete with one another for phosphorylation by CheA_p_, the concentration of phosphorylated CheB increases as [Y_p_] increases and [Y] decreases [35]. However, since phosphorylated CheB has a higher demethylation activity, this tends to lower the activity of the receptor cluster, which in turn tends to *lower* [Y_p_]. In our model, the activity of the receptor cluster is assumed to be the same for wild-type and CheZ mutant cells, and it was chosen such that the concentration of CheY_p_ in adapted wild-type cells is within the working range of the motor. Yet, it is conceivable that because of the negative feedback loop, the activity of the receptor cluster in the adapted state is lower in CheZ mutant cells than in CheZ wild-type cells. This would lower the concentration of CheY_p_ in the CheZ mutant cells and could bring it within the motor's range.

### The response to other attractants

Vaknin and Berg measured not only the response to the addition to serine, but also the response of [Y_p_Z] to changes in aspartate concentration [2]. They found differences in the response between CheZ wild-type cells and CheZ mutant cells when 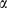-methylaspartate was used as an attractant with 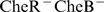 cells expressing *only* the aspartate receptor, Tar. However, no differences were detected when these experiments were repeated with either aspartate or 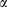-methylaspartate in wild-type cells. In our model, the overall response of [Y_p_Z] to changes in ligand concentration [L] is determined by two independent modules connected in series: 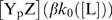. A different attractant only leads to a different response of the receptor cluster, *βk*_0_([L]): the response of 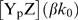 to changes in the activity of the receptor cluster 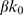 is assumed to be independent of the type of attractant—while 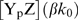 depends upon the nature of CheZ, it is the same for serine and aspartate. Our model would therefore predict that the response to aspartate also differs between CheZ wild-type cells and CheZ mutant cells, in contradiction with the experimental results of Vaknin and Berg [2]. It is conceivable that to explain these observations, the spatial organization of the receptor cluster, in particular the spatial position of CheZ with respect to the aspartate and serine receptors, has to be taken into account, and that a full particle-based model [36],[37] is required to explain the response to both aspartate and serine.

## Methods

The canonical model of the intracellular chemotaxis network of *E. coli* is given by the chemical reactions shown in Equations 1–3. When CheA and CheZ are colocalized at the receptor cluster, the concentration profiles of CheY and CheY_p_ are uniform in space, and the concentrations can be obtained by solving the following chemical rate equations:
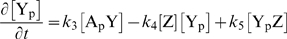
(10)

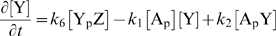
(11)

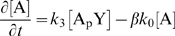
(12)

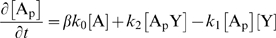
(13)

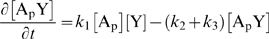
(14)

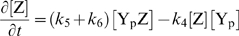
(15)

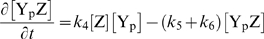
(16)Here, 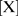 denotes the concentration of species X.

When CheZ cannot bind the receptor cluster and thus diffuses in the cytoplasm, concentration gradients of CheY and CheY_p_ will form. We will assume that the cell is cylindrically symmetric, and we will integrate out the lateral dimensions 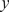 and 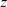. We thus consider a simplified 1-D model, with concentrations as a function of 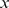. This leads to the following reaction-diffusion equations:
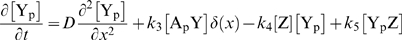
(17)

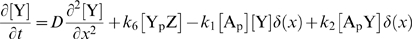
(18)

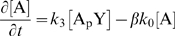
(19)

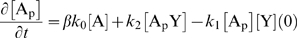
(20)

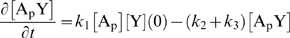
(21)

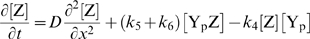
(22)

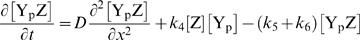
(23)The components CheA, CheA_p_ and CheA_p_CheY are localized at one end of the cell; the unit of their concentrations is the number of molecules per area. The other components diffuse in the cell. Their concentrations, which are in units of number of molecules per volume, depend upon the position 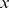 in the cell, where 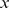 measures the distance from the pole at which CheA, CheA_p_ and CheA_p_CheY are localized; only in Equations 20 and 21 is the 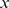 dependence explicitly indicated to emphasize that the CheA_p_-CheY association rate depends on the concentration of CheY at contact. Zero-flux boundary conditions are imposed at both cell ends. The steady-state input-output relations of the network described by Equations 17–23 were obtained numerically by discretizing the system on a (1-D) grid and propagating these equations in space and time until steady state was reached.

The reaction-diffusion equations for the other models described in the main text, i.e. in section *Differential affinity and catalytic activity of CheZ* and section *Cooperativity*, were derived and solved in a similar manner.

## Supporting Information

Text S1 Two independent modules connected in series.
(0.09 MB PDF)

Text S2 Mapping between canonical push-pull network and chemotaxis network.
(0.22 MB PDF)

Text S3 Cooperativity in the phosphatase reactions.
(0.29 MB PDF)
