# Supplementary material for: Differential Affinity and Catalytic Activity of CheZ in E. coli Chemotaxis
Source: PLoS Comput Biol. 2009 May 8;5(5):e1000378. doi: 10.1371/journal.pcbi.1000378 (PMC2673030; doi:10.1371/journal.pcbi.1000378)
Supplement: Text S1 — Two independent modules connected in series. (0.09 MB PDF) [file pcbi.1000378.s001.pdf]

Text S1
Differential affinity and catalytic activity of CheZ in E. coli *chemotaxis*

S.B. van Albada and P.R. ten Wolde
(Dated: February 9, 2009)

Two independent modules connected in series

The experiments of Vaknin and Berg [1] allow us to verify whether the receptor cluster and the intracellular signaling pathway are indeed two independent modules connected in series. Vaknin and Berg performed experiments on four bacterial strains: wild type CheR$^+$CheB$^+$CheZ cells, CheR$^-$CheB$^-$CheZ cells lacking CheR and CheB, CheR$^+$CheB$^+$CheZ* cells with mutant CheZ proteins that cannot bind the receptor cluster, and CheR$^-$CheB$^-$CheZ* cells lacking CheR and CheB and with CheZ* (mutated CheZ). As discussed in the main text, we assume that $\beta k_0[L]$ is the same for wild type and CheZ mutant cells, but not for wild type and *cheRcheB* cells; in contrast, $[\mathrm{Y_pZ}](\beta k_0)$ is the same for wild type and *cheRcheB* cells, but not for wild type and CheZ mutant cells. If the receptor cluster and the chemotaxis network are two independent modules connected in series, then it should be possible to describe the response curve $[\mathrm{Y_pZ}]([\mathrm{L}])$ of each of the four strains using two of the following response curves: $[\mathrm{Y_pZ}]^{Z^{\mathrm{wt}}}(\beta k_0)$ or $[\mathrm{Y_pZ}]^{Z^*}(\beta k_0)$, and $\beta k_0^{R^+B^+}([\mathrm{L}])$ or $\beta k_0^{R^-B^-}([\mathrm{L}])$. In other words, all four response curves measured in [1] should be of the composite form $[\mathrm{Y_pZ}]^{\{Z^*,Z^{wt}\}}\left(\beta k_0^{RB\pm}([\mathrm{L}])\right)$.

Figure 1 shows that the results of Vaknin and Berg [1] are consistent with the above assumptions. The figure shows $[\mathrm{Y_pZ}]$ in cells containing wild-type CheZ as a function of $[\mathrm{Y_pZ}]$ in cells containing mutant CheZ, both for *cheRcheB* cells and cells containing CheR and CheB. These figures are obtained from Figures 5A and 5C of Ref. [1], which show the effect of mutating CheZ on the renormalized FRET response, (FRET([L])/FRET([L]=0)), for *cheRcheB* cells and cells containing CheR and CheB, respectively. The FRET signal is proportional to $[\mathrm{Y_pZ}]$, which means that these figures are proportional to $[\mathrm{Y_pZ}]([\mathrm{L}])$. Since we assume that mutating CheZ has no influence on the response of the receptor cluster to ligand, $\beta k_0([\mathrm{L}])$, each ligand concentration corresponds to a unique activity of the receptor cluster, for a given type of cells (meaning either *cheRcheB* cells or cells containing the (de)methylation enzymes). Therefore, it is meaningful to plot the FRET signal of cells with wild-type CheZ as a function of the FRET signal of CheZ mutant cells at the same ligand concentration (and hence activity of the receptor cluster), both for *cheRcheB* cells (Fig.5A of Ref. [1]) and cells containing CheR and CheB (Fig.5C of Ref. [1]). If, according to our assumptions, $\beta k_0[\mathrm{L}]$ only depends upon the presence of CheR and CheB while $[\mathrm{Y_pZ}]$ only depends upon the nature of CheZ, it should be possible to scale these curves such as to make them overlap. To be more precise, in Figure 1 there are 4 different amplitudes, corresponding to $[\mathrm{Y_pZ}]([\mathrm{L}]=0)$ for wild-type cells, cells with CheR and CheB and with mutant CheZ, *cheRcheB* cells with wild-type CheZ, and *cheRcheB* cells with mutant CheZ; the two amplitudes of CheR$^+$CheB$^+$ cells, correspond, respectively, to the x- and y-coordinate of the top-right circle, while the two amplitudes of *cheRcheB* cells correspond to the top-right black square. Because no absolute FRET signal is given in Ref. [1], each of the two curves in Fig. 1, can be scaled independently in both the x- and y-direction, such as to make the curves overlap. Figure 1 is obtained by scaling the FRET signal for the wild-type (CheR$^+$CheB$^+$CheZ) cells by a factor

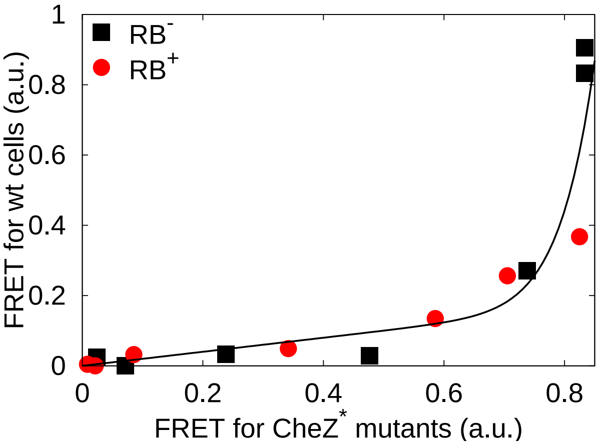

FIG. 1: The relation between the four FRET signals of Figs. 5a and 5c in [1]. FRET signals of bacterial strains that differ only in the type of phosphatase—wild-type CheZ versus a non-localizing mutant–are plotted as a function of each other. This was done both for strains lacking the adaptation proteins CheR and CheB (squares) and for strains with CheR and CheB (circles). The FRET signals on the vertical and horizontal axes are linked via equal concentrations of attractant in the reponse curves. Because of the decoupling of the response of the intracellular signal transduction pathway from the response of the receptor cluster, equal concentrations of attractant correspond to equal activities of the receptor cluster if the CheRCheB states are the same, and equal concentrations of $[\mathrm{Y_pZ}]$ correspond to equal activities of the receptor cluster if the same type of phosphatase is present. As a consequence, the two curves should overlap (see main text). This could be achieved by rescaling only the FRET signal for the wild-type bacterium by a factor 0.5.

0.5. It is seen that the curves for $CheR^+CheB^+$ cells and $CheR^-CheB^-$ cells indeed overlap. This supports the idea that the receptor cluster and the intracellular pathway are two independent modules connected in series.

Figure 1 allows us to rescale the data of Figures 5A and 5C of Ref. [1] to obtain $[Y_pZ]$ as a function of [L]. The result is shown in Figure 1A of the main text, where the maximum $[Y_pZ]$, obtained for $CheR^-CheB^-CheZ$ cells at $[L] = 0$, was set to the total CheZ concentration as reported in [2]. These response curves impose strong constraints on any model that hopes to explain the response of $CheY_pCheZ$ to the addition of serine.

[1] Vaknin A, Berg HC (2004) Single-cell FRET imaging of phosphatase activity in the *Escherichia coli* chemotaxis system. Proc Natl Acad Sci USA 101:17072–17077.

[2] Sourjik V, Berg HC (2002) Binding of the *Escherichia coli* response regulator CheY to its target measured in vivo by fluorescence resonance energy transfer. Proc Natl Acad Sci USA 99:12669 – 12674.
