# Supplementary material for: Differential Affinity and Catalytic Activity of CheZ in E. coli Chemotaxis
Source: PLoS Comput Biol. 2009 May 8;5(5):e1000378. doi: 10.1371/journal.pcbi.1000378 (PMC2673030; doi:10.1371/journal.pcbi.1000378)
Supplement: Text S2 — Mapping between canonical push-pull network and chemotaxis network. (0.22 MB PDF) [file pcbi.1000378.s002.pdf]

# Text S2
## *Differential affinity and catalytic activity of CheZ in* E. coli *chemotaxis*

S.B. van Albada and P.R. ten Wolde
(Dated: February 9, 2009)

## Mapping between canonical push-pull network and chemotaxis network

The canonical model for the cytosolic signal transduction pathway in the *E. coli* chemotaxis system is given by Eqns. 1-3 of the main text. The system is very similar to the canonical push-pull network, given by the following chemical reactions:

$$\mathrm{E_a + X \underset{k_2}{\overset{k_1}{\rightleftharpoons}} E_aX \overset{k_3}{\rightarrow} E_a + X^*} \quad (1)$$

$$\mathrm{E_d + X^* \underset{k_5}{\overset{k_4}{\rightleftharpoons}} E_dX^* \overset{k_6}{\rightarrow} E_d + X} \quad (2)$$

In steady state, a direct mapping is possible between both networks. This can be seen by comparing the chemical rate equations and the total concentrations for the two systems:

$$\underline{\text{Chemotaxis network}} \quad \text{vs.} \quad \underline{\text{Canonical push-pull network}}$$

$$\mathrm{\frac{d[Y]}{dt} = k_2[A_pY] + k_6[Y_pZ] - k_1[A_p][Y] \Leftrightarrow \frac{d[X]}{dt} = k_2[E_aX] + k_6[E_dX^*] - k_1[E_a][X]} \quad (3)$$

$$\mathrm{\frac{d[Y_p]}{dt} = k_5[Y_pZ] + k_3[A_pY] - k_4[Z][Y_p] \Leftrightarrow \frac{d[X^*]}{dt} = k_5[E_dX^*] + k_3[E_aX] - k_4[E_d][X^*]} \quad (4)$$

$$\mathrm{\frac{d[Y_pZ]}{dt} = k_4[Z][Y_p] - (k_5 + k_6)[Y_pZ] \Leftrightarrow \frac{d[E_dX^*]}{dt} = k_4[E_d][X^*] - (k_5 + k_6)[E_dX^*]} \quad (5)$$

$$\mathrm{\frac{d[Z]}{dt} = -\frac{d[Y_pZ]}{dt} \Leftrightarrow \frac{d[E_d]}{dt} = -\frac{d[E_dX^*]}{dt}} \quad (6)$$

$$\mathrm{\frac{d[A_pY]}{dt} = k_1[A_p][Y] - (k_2 + k_3)[A_pY] \Leftrightarrow \frac{d[E_aX]}{dt} = k_1[E_a][X] - (k_2 + k_3)[E_aX]} \quad (7)$$

$$\mathrm{\frac{d[A_p]}{dt} = -\frac{d[A_pY]}{dt} - \frac{d[A]}{dt} \Leftrightarrow \frac{d[E_a]}{dt} = -\frac{d[E_aX]}{dt}} \quad (8)$$

$$\mathrm{\frac{d[A]}{dt} = k_3[A_pY] - \beta k_0[A]} \quad (9)$$

$$\mathrm{[Y]_T = [Y] + [Y_p] + [Y_pZ] + [A_pY] \Leftrightarrow [X]_T = [X] + [X^*] + [E_aX] + [E_dX^*]} \quad (10)$$

$$\mathrm{[Z]_T = [Z] + [Y_pZ] \Leftrightarrow [E_d]_T = [E_d] + [E_dX^*]} \quad (11)$$

$$\mathrm{[A_p]_T = [A_p] + [A_pY] = [A]_T - [A] \Leftrightarrow [E_a]_T = [E_a] + [E_aX]} \quad (12)$$

As $\frac{dA}{dt}$ in Eqn. 8 equals zero in steady state, it follows that the steady state of the chemotaxis model with total concentrations $[Y]_T$, $[Z]_T$ and $[A_p]_T$ is identical to the steady state of a push-pull network with total concentrations $[X]_T$, $[E_d]_T$ and $[E_a]_T$, respectively. The remaining concentration of unphosphorylated A is then $[A] = [A]_T - [A_p]_T$ (Eqn. 12) and $\beta k_0$ equals $k_3[A_pY][A]^{-1}$ (Eqn. 9). This mapping also holds for non-uniform networks with any spatial arrangement of the enzymes, e.g., with the activating enzyme localized at one end of the cell and the deactivating enzyme freely diffusive.

The canonical chemotaxis model is thus simply a push-pull network of which the concentration of activating enzyme $[E_a]_T$ is tuned via the parameter $\beta k_0$ while the concentration of deactivating enzyme $[E_d]_T$ is kept constant. The steady state of a push-pull network with given substrate and enzyme concentrations is fully determined by the ratio of the catalytic activities $k_3/k_6$ and the Michaelis-Menten constants $K_{M,a} \equiv (k_2+k_3)/k_1$ and $K_{M,d} \equiv (k_5 + k_6)/k_4$, as can be verified from the above

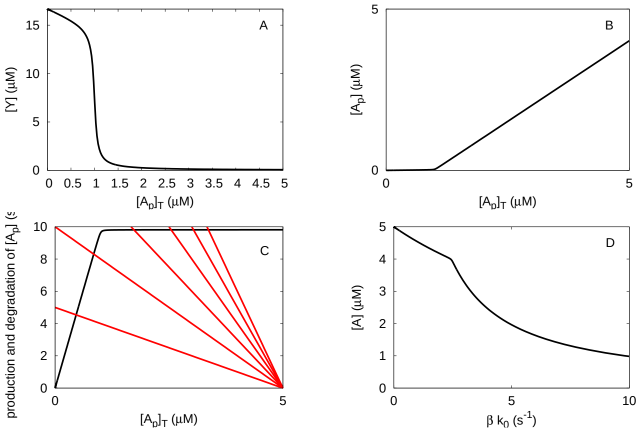


FIG. 1: Mapping of a *symmetric* network with a topology of that of the canonical *E. coli* chemotaxis network (Equations 1-3 of main text) onto a symmetric canonical push-pull network (Equations 1-2), for networks that are in the *zero-order* regime. $k_1 = k_4 = 36\ \mu\mathrm{M}^{-1}\mathrm{s}^{-1}$; $k_3 = k_6 = 10\ \mathrm{s}^{-1}$ ($K_\mathrm{M} = 0.28\ \mu$M); $S_\mathrm{T} = [\mathrm{Y}]_\mathrm{T} = 16.7\ \mu$M; $[\mathrm{E_d}]_\mathrm{T} = [\mathrm{Z}]_\mathrm{T} = 1\ \mu$M; $[\mathrm{A}]_\mathrm{T} = 5\mu$M. In panel C: the black line is given by the right-hand side of Eq. 14, i.e. $k_1[\mathrm{A_p}]([\mathrm{A_p}]_\mathrm{T})[\mathrm{Y}]([\mathrm{A_p}]_\mathrm{T})$; the red lines are given by the left-hand side of Eq. 14, i.e. $\beta k_0([\mathrm{A}]_\mathrm{T} - [\mathrm{A_p}]_\mathrm{T})$; the different red lines correspond to different values of $\beta k_0$ : $\beta k_0 = 1, 2, 3, 4, 5, 6\,\mathrm{s}^{-1}$. The intersection of the black and red curves yields $[\mathrm{A_p}]_\mathrm{T}$ as a function of $\beta k_0$. When $[\mathrm{A_p}]_\mathrm{T}$ is determined, the state of the system is fully determined.

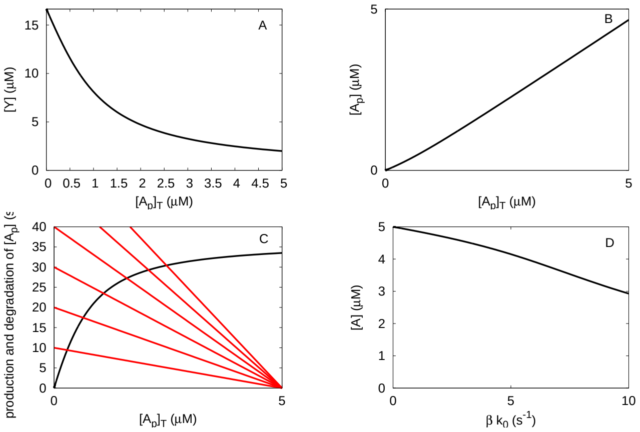


FIG. 2: Mapping of a *symmetric* network with a topology of that of the canonical *E. coli* chemotaxis network (Equations 1-3 of main text) onto a symmetric canonical push-pull network (Equations 1-2), for networks that are in the *linear* regime. The different red lines correspond to different values of $\beta k_0$ : $\beta k_0 = 2, 4, 6, 8, 10, 12\,\mathrm{s}^{-1}$. $k_1 = k_4 = 3.6\ \mu\mathrm{M}^{-1}\mathrm{s}^{-1}$; $k_3 = k_6 = 100\ \mathrm{s}^{-1}$ ($K_\mathrm{M} = 28\ \mu$M); $S_\mathrm{T} = [\mathrm{Y}]_\mathrm{T} = 16.7\ \mu$M; $[\mathrm{E_d}]_\mathrm{T} = [\mathrm{Z}]_\mathrm{T} = 1\ \mu$M; $[\mathrm{A}]_\mathrm{T} = 5\mu$M.

system of equations. Therefore, the steady state of the chemotaxis model is fully determined by the same combination of parameters, together with $\beta \mathrm{k}_0$. In particular, we can, without loss of generality, set $k_2$ and $k_5$ equal to zero—they only affect the response via their effect on the Michaelis-Menten constants.

It turns out that this mapping between a canonical push-pull network and a network with a topology of that of the chemotaxis system, is particularly useful for understanding the response of $[\mathrm{Y_p}]$ and $[\mathrm{Y_pZ}]$ and hence the FRET signal to changes in the activity of the receptor cluster $\beta k_0$. This is illustrated in Figures 1 and 2 for a spatially uniform network in the zero-order and linear regime, respectively; as discussed in [3], this also corresponds to a push-pull network in which the enzymes are colocalized at one end of the cell. In steady state, the chemotaxis network obeys the following relation:

$$\beta k_0[\mathrm{A}] = k_1[\mathrm{A_p}][\mathrm{Y}], \tag{13}$$

where we have assumed that $k_2 = 0$. The idea is now that $[\mathrm{A_p}]$ and $[\mathrm{Y}]$ are fully determined by the total concentration of phosphorylated CheA, $[\mathrm{A_p}]_\mathrm{T} \equiv [\mathrm{A_p}] + [\mathrm{A_pY}]$, as in a canonical push-pull network: $[\mathrm{A_p}] \equiv [\mathrm{A_p}]([\mathrm{A_p}]_\mathrm{T})$ and $[\mathrm{Y}] \equiv [\mathrm{Y}]([\mathrm{A_p}]_\mathrm{T})$. The functions $[\mathrm{Y}]([\mathrm{A_p}]_\mathrm{T})$ and $[\mathrm{A_p}]([\mathrm{A_p}]_\mathrm{T})$ can be obtained analytically [4], and they are shown in Figs. 1-2A and 1-2B, respectively. The concentration of $[\mathrm{A_p}]_\mathrm{T}$, in turn, is controlled by the value of $\beta k_0$. To obtain $[\mathrm{A_p}]_\mathrm{T}$ as a function of $\beta k_0$, we rewrite the above equation as

$$\beta k_0\left([\mathrm{A}]_\mathrm{T} - [\mathrm{A_p}]_\mathrm{T}\right) = k_1[\mathrm{A_p}]([\mathrm{A_p}]_\mathrm{T})[\mathrm{Y}]([\mathrm{A_p}]_\mathrm{T}), \tag{14}$$

where $[\mathrm{A}]_\mathrm{T} \equiv [\mathrm{A}] + [\mathrm{A_p}]_\mathrm{T}$ is the total concentration of CheA. This equation can now be solved for $[\mathrm{A_p}]_\mathrm{T}$ as a function of $\beta k_0$. The behavior of the solution can be understood by plotting the left-hand side and the right-hand side of the above equation separately, as is illustrated in Figures 1C and 2C for networks in the zero-order and linear regime, respectively; the intersection yields the value of $[\mathrm{A_p}]_\mathrm{T}$. The panels D in these figures show $[\mathrm{A}] = [\mathrm{A}]_\mathrm{T} - [\mathrm{A_p}]_\mathrm{T}$ as a function of $\beta k_0$. Since all the other concentrations $[\mathrm{Y}]$, $[\mathrm{Y_p}]$, $[\mathrm{A_pY}]$, and $[\mathrm{Y_pZ}]$ are determined by $[\mathrm{A_p}]_\mathrm{T}$, the state of the system is now fully specified.

## The effect of phosphatase localization within the canonical chemotaxis model

In Ref. [1], dose response curves were measured for the binding of $\mathrm{Y_p}$ to Z as a function of the extracellular concentration of the ligand (L) serine, both for $\mathrm{CheR^+CheB^+}$ and $\mathrm{CheR^-CheB^-}$ cells. In what follows, we focus exclusively on $\mathrm{CheR^+CheB^+}$ cells. The concentration of the $\mathrm{Y_pZ}$ complex was measured in [1] via Fluorescence Resonance Energy Transfer (FRET). Figure 5c of Ref. [1] shows that there is a large difference between the dose response curves of wild-type cells as compared to those of CheZ mutant cells, which contain a non-localizing version of the phosphatase CheZ. In the following, we will verify whether the canonical chemotaxis model can explain the experimentally measured difference in the response of wild-type cells and CheZ mutant cells [1]. We show in the main text (Fig. 2) that

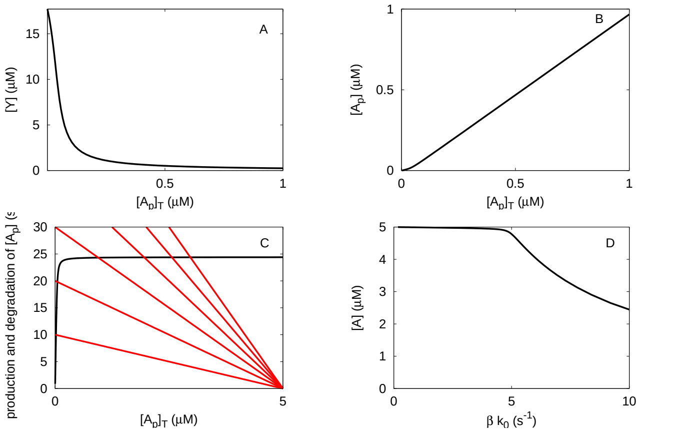


FIG. 3: Mapping of the canonical *E. coli* chemotaxis network (Equations 1-3 of main text) onto a push-pull network (Equations 1-2). The black line corresponds to the rate of $\mathrm{CheA_p}$ production as given by the right-hand side of Eq. 14, while the red lines correspond to the "decay" rate of $\mathrm{CheA_p}$ as given by the left-hand side of Eq. 14; each red line corresponds to a different value of $\beta k_0$: from left to right, $\beta k_0 = 2,4,6,8,10,12\,\mathrm{s}^{-1}$. The intersection of the black and red lines yield $[\mathrm{A_p}]_\mathrm{T}$ in steady state. Please note that since the rate of $\mathrm{CheA_p}$ deactivation (black line) initially increases rapidly with $[\mathrm{A_p}]_\mathrm{T}$, [A] as a function of $\beta k_0$ is essentially constant and given by $[\mathrm{A}] \approx [\mathrm{A}]_\mathrm{T}$ for low values of $\beta k_0$, as shown in panel D. If $[\mathrm{A}] \approx [\mathrm{A}]_\mathrm{T}$, $[\mathrm{Y_pZ}]$ increases linearly with the activity of the receptor cluster, $\beta k_0$, since in steady state $\beta k_0[\mathrm{A}] = k_6[\mathrm{Y_pZ}]$ (see, e.g., Fig. 4). $k_1 = 100\mu\mathrm{M}^{-1}\mathrm{s}^{-1}$; $k_3 = 750\ \mathrm{s}^{-1}$; $k_4 = 5\ \mu\mathrm{M}^{-1}\mathrm{s}^{-1}$; $k_6 = 30\ \mathrm{s}^{-1}$; $[\mathrm{S}]_\mathrm{T} = [\mathrm{Y}]_\mathrm{T} = 17.9\ \mu M$; $[\mathrm{E_d}]_\mathrm{T} = [\mathrm{Z}]_\mathrm{T} = 1.1\ \mu\mathrm{M}$; $[\mathrm{A}]_\mathrm{T} = 5\mu\mathrm{M}$.

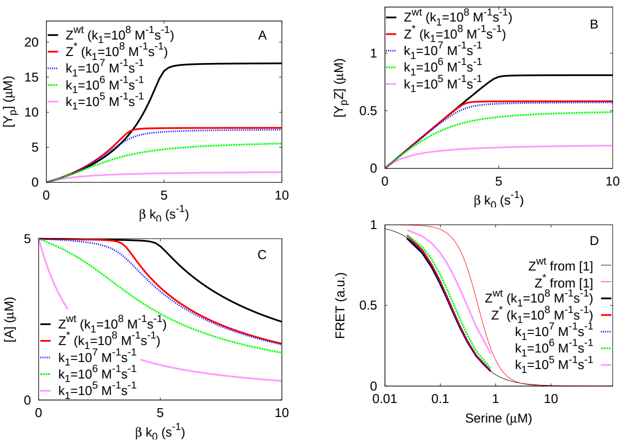


FIG. 4: Effect of varying $k_1$ on the response of the canonical chemotaxis model (Equations 1-3 of the main text). The thick black line corresponds to the predicted response of wild-type cells, the parameters of which are kept constant (except $\beta k_0$); the other thick lines correspond to predicted response curves of CheZ mutant cells, where each line corresponds to a different value of $k_1$. Shown are the response of $[\mathrm{Y_p}]$ (A), $[\mathrm{Y_pZ}]$ (B), [A] (C) to changes in the activity of the receptor cluster $\beta k_0$ and the FRET signal as a function of serine concentration (D). By construction (see text), the predicted FRET response of the wild-type cells (thick black line) coincides with the measured response [1] (thin black line). Please also note that for lower CheA-CheY$_\mathrm{p}$ association rates ($k_1 = 10^5\mathrm{M}^{-1}\mathrm{s}^{-1}$, magenta line) in CheZ mutant cells, the predicted FRET response shifts in the direction of the one measured for CheZ mutant cells (thin red line) [1]. The baseline parameters are $k_1 = 10^8\ \mathrm{M}^{-1}\mathrm{s}^{-1}$, $k_3 = 750\ \mathrm{s}^{-1}$, $k_4 = 5\cdot 10^6\ \mathrm{M}^{-1}\mathrm{s}^{-1}$, $k_5 = 0.5\ \mathrm{s}^{-1}$, $k_6 = 30\ \mathrm{s}^{-1}$, $[\mathrm{Z}]_\mathrm{T} = 1.1\mu\mathrm{M}$, $[\mathrm{A}]_\mathrm{T} = 5\mu\mathrm{M}$ and $[\mathrm{Y}]_\mathrm{T} = 17.9\mu\mathrm{M}$ ([2]) and $D = 5\ \mu\mathrm{m}^2\mathrm{s}^{-1}$.

the canonical model cannot explain this difference in response if the only difference between wild-type cells and CheZ mutant cells is the spatial distribution of CheZ. We now address the question whether allowing also one of the other parameters (rate constants, concentrations) to be different between wild-type and CheZ mutant cells does make it possible to fit the data of Vaknin and Berg [1]. In practice, we try to fit the dose response curve of FRET vs. [serine] for both wild-type and CheZ mutant cells simultaneously, by varying the parameters for the mutant bacterium, while keeping the parameters for the wild-type bacterium fixed. The dose response curves of FRET vs. [serine] are obtained by combining the response of $[\mathrm{Y_pZ}]$ to the receptor-cluster activity $\beta k_0$ and the response of $\beta k_0$ to the concentration of added ligand, as discussed in more detail below.

Figures 4-7 show the effect of individually varying the rate constants $k_1$, $k_3$, $k_4$ and $k_6$ of the canonical model of the intracellular chemotaxis network (Equations 1-3 of the main text). For every parameter set we show the response of: A) $[\mathrm{Y_p}]$ as a function of the receptor-cluster activity $\beta k_0$; B) $[\mathrm{Y_pZ}]$ as a function of $\beta k_0$; C) [A] as a function of $\beta k_0$; D) the FRET signal as a function of added ligand (serine). Every plot shows the result for the wild-type cell with colocalized CheA and CheZ and with the baseline parameter set (black line), together with the response curves of mutant cells with diffusive CheZ, where each curve corresponds to a different value of the rate constant that is varied. The calculations were repeated for different concentrations $[\mathrm{A}]_\mathrm{T}$ and $[\mathrm{Y}]_\mathrm{T}$ with similar results (data not shown).

Figures 4C-7C show [A] as a function of $\beta k_0$. It is seen that for low values of $\beta k_0$, [A] is essentially constant, and given by $[\mathrm{A}] \approx [\mathrm{A}]_\mathrm{T}$. This is an important observation. In steady state, $\beta k_0[\mathrm{A}] = k_6[\mathrm{Y_pZ}]$ for the canonical model of the intracellular chemotaxis network. Hence, when [A] is constant, $[\mathrm{Y_pZ}]$ varies linearly with $\beta k_0$. Since the FRET signal is proportional to $[\mathrm{Y_pZ}]$, also the FRET signal varies linearly with $\beta k_0$, when [A] is constant. If the FRET signal is proportional to $\beta k_0$, then the *renormalized* FRET response is fully determined by the activity of the receptor cluster: $\mathrm{FRET}([\mathrm{L}])/\mathrm{FRET}([\mathrm{L}]=0) = \beta k_0([\mathrm{L}])/\beta k_0([\mathrm{L}]=0)$; it no longer depends upon parameters of the intracellular network. The observation of $[\mathrm{A}] \approx [\mathrm{A}]_\mathrm{T}$ is thus important, because a) it would justify the commonly made assumption that the renormalized FRET response reflects the activity of the receptor cluster; b) it would

mean that the canonical model cannot explain the difference in FRET response between wild-type cells and CheZ mutant cells, since the spatial distribution of CheZ is assumed to only affect the response of the intracellular network and not that of the receptor cluster.

Figure 4C shows that over the concentration range of interest (corresponding to $\beta k_0 < \beta k_0^{\rm ns} \approx 3{\rm s}^{-1}$), $[{\rm A}] \approx [{\rm A}]_{\rm T}$ is fairly constant in wild-type cells and CheZ mutant cells with the baseline parameter set. This would justify the assumption that the renormalized FRET response is a useful measure for the activity of the receptor cluster. However, by the same token, it also means that the canonical model cannot explain the experiments by Vaknin and Berg [1].

Figures 4A–7A and 4B–7B show the response of $[{\rm Y_p}]$ and $[{\rm Y_pZ}]$ as a function of $\beta k_0$. There exists a simple relation between the curves $[{\rm Y_pZ}](\beta {\rm k}_0)$ and $[{\rm Y_p}](\beta {\rm k}_0)$. In steady state, $[{\rm Y_p}][{\rm Z}] = {\rm K_{MZ}}[{\rm Y_pZ}]$, where $K_{\rm MZ} = (k_5 + k_6)/k_4$. Since, $[{\rm Z}] = [{\rm Z}]_{\rm T} - [{\rm Y_pZ}]$, $[{\rm Y_p}]$ can be expressed in terms of $[{\rm Y_pZ}]$ as $[{\rm Y_p}] = {\rm K_{MZ}}[{\rm Y_pZ}]/([{\rm Z}]_{\rm T} - [{\rm Y_pZ}])$. This relation immediately gives the functional form of $[{\rm Y_p}](\beta k_0)$ when $[{\rm Y_pZ}]$ depends linearly on $\beta k_0$.

We now address the question *why* [A] as a function of $\beta k_0$ is initially constant, and then suddenly decreases. To this end, we will exploit the mapping between the *E. coli* chemotaxis network and the canonical push-pull network, as illustrated in Fig. 3. In steady state, Equation 13 and Equation 14 hold. Figure 3C shows the rate of $\rm CheA_p$ production and $\rm CheA_p$ deactivation, corresponding to the left-hand side (lhs) and right-hand side (rhs) of Equation 14, respectively, as a function of $[{\rm A_p}]_{\rm T}$. The rate of $\rm CheA_p$ deactivation (rhs) is given by $k_1[{\rm A_p}]([{\rm A_p}]_{\rm T})[{\rm Y}]([{\rm A_p}]_{\rm T})$. As shown in Fig. 3B, for the *E. coli* network $[{\rm A_p}] \approx [{\rm A_p}]_{\rm T}$. Hence, the slope of the rhs is given by $k_1[{\rm Y}]([{\rm A_p}]_{\rm T})$. Fig. 3A shows the concentration of CheY as a function of the total $\rm CheA_p$ concentration, i.e. $[{\rm Y}]([{\rm A_p}]_{\rm T})$. It is seen that [Y] is high for low values of $[{\rm A_p}]_{\rm T}$; this explains the large initial slope of $k_1[{\rm A_p}][{\rm Y}]$ as a function of $[{\rm A_p}]_{\rm T}$ in Fig. 3C. Figure 3A also shows that as $[{\rm A_p}]_{\rm T}$ is increased, [Y] decreases strongly. This explains the strong drop in the slope of $k_1[{\rm A_p}][{\rm Y}]$ (rhs) as $[{\rm A_p}]_{\rm T}$ is increased. Because $k_1[{\rm A_p}][{\rm Y}]$ (rhs Eq. 14) initially rises rapidly with $[{\rm A_p}]_{\rm T}$ and then levels off abruptly, the intersection with the curve $\beta k_0[{\rm A}]$ (lhs Eq. 13), which determines the steady state, initially occurs for very low values of $[{\rm A_p}]_{\rm T}$ as $\beta k_0$ is increased from zero. Only when the activity of the receptor cluster, $\beta k_0$, is such that the total $\rm CheA_p$ concentration becomes large enough to decrease [Y], does $[{\rm A_p}]$ increase and [A] decrease, as shown in Fig. 3D. Put differently, initially the $\rm CheA_p$ molecules that are produced, immediately react with CheY molecules to yield CheA molecules again. This keeps the concentration of $\rm CheA_p$ low. However, in this process, the concentration of CheY does decrease, and this reduces the rate at which $\rm CheA_p$ molecules are dephosphorylated. At some point, [Y], and hence the rate of $\rm CheA_p$ dephosphorylation, has decreased so much, that the concentration of $\rm CheA_p$ will rapidly rise.

Figs. 1 and 2 show the results for a symmetric push-pull network in the zero-order and first-order regime, respectively. For the zero-order network, it is seen that $[{\rm A}](\beta k_0)$ has two distinct regimes. The first corresponds to the regime in which the phosphatase activity is larger than the kinase activity, and [Y] is large (Fig. 1A); note that since the network is zero-order, also the concentration of [A] is low (Fig. 1B). Because [Y] is high in this regime, the initial slope of $k_1[{\rm A_p}][{\rm Y}]$ as a function of $[{\rm A_p}]_{\rm T}$ is large (Fig. 1C). The second regime corresponds to the one in which the kinase activity exceeds the phosphatase activity; $[{\rm Y_p}]$ is large and [Y] is low; because [Y] is now very low, the slope is essentially reduced to zero (Fig. 1C). The situation differs markedly for a push-pull network in the linear regime. In this regime, the concentration of [Y] changes gradually as a function of $[{\rm A_p}]_{\rm T}$ (Fig. 2A) and this leads to a gradual change in the slope of $k_1[{\rm A_p}][{\rm Y}]$ as a function of $[{\rm A_p}]_{\rm T}$ (Fig. 2C). This gradual change in the slope manifests itself as a gradual change in $[{\rm A}](\beta k_0)$ (Fig. 2D).

We are now in a position to understand how the response curves change as the rate constants are varied. As $k_1$ is decreased, the push-pull network becomes more linear, as a result of which the concentration of $[{\rm Y_p}]$ decreases more gradually as $[{\rm A_p}]_{\rm T}$ increases. Moreover, as $k_1$ is decreased, the rate at which $\rm CheA_p$ molecules are dephosphorylated decreases. These two effects combine to yield a more gradual change in the rate of $\rm CheA_p$ deactivation (the rhs of Eq. 14) as a function of $[{\rm A_p}]_{\rm T}$; as seen for the symmetric push-pull network in the linear regime (Fig. 2C), such a gradual change in $k_1[{\rm A_p}][{\rm Y}]$ as a function of $[{\rm A_p}]_{\rm T}$, means that [A] starts to decrease at lower values of $\beta k_0$ and then does so more gradually (see Fig. 4). When $k_3$ is decreased, the network enters the zero-order regime more deeply, and the response becomes similar to that of the symmetric push-pull network in the zero-order regime (compare Figs. 1 and 5). When $k_4$ is decreased, [Y] decreases at lower values of $[{\rm A_p}]_{\rm T}$ and does so more gradually, since the network becomes more linear; consequently, [A] starts to decrease at lower values of $\beta k_0$ (Fig. 6C). Lastly, when $k_6$ is decreased, [Y] decreases more sharply for lower values of $[{\rm A_p}]_{\rm T}$. As a result, $[{\rm A}](\beta k_0)$ starts to decrease at lower values of $\beta k_0$ and then does so more strongly (Fig. 7C). Please note that in all cases, when [A] is no longer constant and equal to $[{\rm A}]_{\rm T}$, $[{\rm Y_pZ}](\beta k_0)$ is no longer a straight line, but becomes a concave function (Figs. 4B–7B). As discussed in the main text, such a concave function for CheZ mutant cells over the concentration range of interest, could make it possible to simultaneously fit the measured dose-response curves for wild-type and CheZ mutant cells [1].

To show the degree of agreement with experiment that can be obtained, we present in Figures 4D–7D the predictions of the canonical model for the FRET signal as a function of ligand concentration for both wild-type and CheZ mutant cells. These curves are obtained as follows. First, we note that $[{\rm Y_pZ}]([{\rm L}])$ is given by $[{\rm Y_pZ}](\beta k_0([{\rm L}]))$. For wild-type cells, $[{\rm Y_pZ}]$ is linear in $\beta k_0$, which means

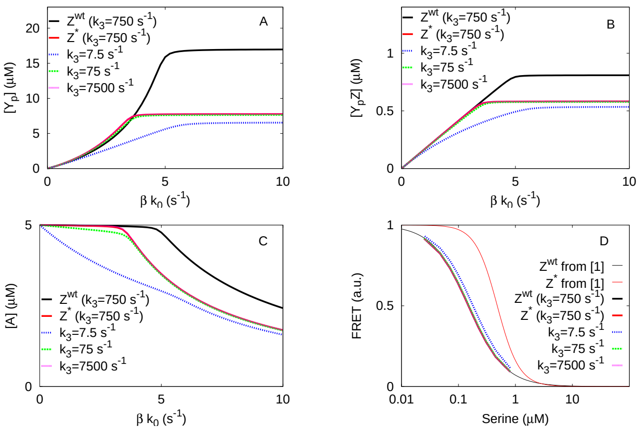


FIG. 5: Effect of $k_3$ on the response of the canonical chemotaxis model (Equations 1-3 of the main text). Shown are the response of $[\mathrm{Y_p}]$ (A), $[\mathrm{Y_pZ}]$ (B), [A] (C) to changes in the activity of the receptor cluster $\beta k_0$ and the FRET signal as a function of serine concentration (D). The baseline parameters are given in Fig. 4.

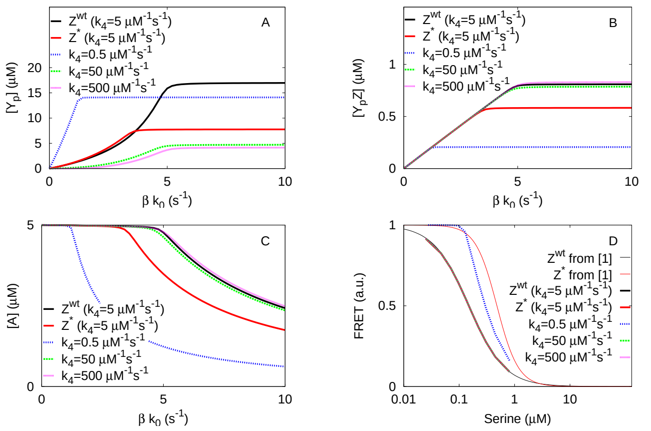


FIG. 6: Effect of $k_4$ on the response of the canonical chemotaxis model (Equations 1-3 of the main text). Shown are $[\mathrm{Y_p}]$ (A), $[\mathrm{Y_pZ}]$ (B), [A] (C) as a function of $\beta k_0$, and the FRET signal as a function of serine concentration (D). Please note that for a lower CheZ-CheY$_\mathrm{p}$ association rate ($k_4 = 10^5$ M$^{-1}$s$^{-1}$, blue line) in CheZ mutant cells, the predicted FRET response of CheZ mutant cells agrees fairly well with the experimentally measured one (thin red line) [1]. The baseline parameters are given in Fig. 4.

that apart from a proportionality factor, $\beta k_0([\mathrm{L}])$ is given by $[\mathrm{Y_pZ}]([\mathrm{L}])$. The latter is obtained up to a scaling factor by the FRET data in Fig. 5c of [1], which can be fitted to a Hill function. Hence, $\beta k_0([\mathrm{L}])$ can be described by a Hill function $\beta k_0([\mathrm{L}]) = \frac{C_1}{1+([\mathrm{L}]/K_\mathrm{D})^{n_\mathrm{H}}}$, where $K_\mathrm{D}$ and $n_\mathrm{H}$ can directly be obtained from the fit to the FRET data. The constant $C_1$ is given by the value of $\beta k_0$ at zero ligand concentration, i.e. in the non-stimulated state: $C_1 = \beta k_0^{\mathrm{ns}}$; we chose the constant such that $[\mathrm{Y_p}](\beta k_0^{\mathrm{ns}}) \approx 3\mu$M, which is in the middle of the working range of the motor. Note that, by construction, the FRET response of wild-type cells, as predicted by the canonical model, agrees with that observed experimentally. The FRET curves for the CheZ mutant cells can now be obtained by combining the computed $[\mathrm{Y_pZ}](\beta k_0)$ for these cells with $\beta k_0([\mathrm{L}])$, which is assumed to be the same for both wild-type cells and CheZ mutant cells; thus, not only $K_\mathrm{D}$ and $n_\mathrm{H}$ are the same, but also $\beta k_0^{\mathrm{ns}}$ and hence $C_1$; as discussed in the main text, this relies on the assumption that there is no feedback from CheY to the activity of the receptor cluster, which could affect the value of $\beta k_0$ in the non-stimulated state.

The results of this procedure are shown in Figs. 4D-7D. For the wild-type cell, the predicted dose-response curve indeed coincides with the experimental curve as measured in [1], while for the mutant cells the predicted response curves typically deviate from those measured experimentally. A good simultaneous fit to the dose-response curves of the wild-type and CheZ mutant cells can be obtained by assuming a lower value of the catalyic rate $k_6$ for the CheZ mutant cells (see Fig. 7D). Both the lower sensitivity for the mutant cells as well as the increased sharpness of the dose resonse curve are reproduced if the catalytic activity of CheZ, $k_6$, is approximately ten times lower for the mutant cells than for the wild-type cells.

Although the fit to the dose response curves is good, it can also be seen from Figs. 7A and 7B that the concentration of CheY$_\mathrm{p}$ and the concentration of CheY$_\mathrm{p}$CheZ are at their maximum values for the mutant cells when they are in their non-stimulated state, i.e. when $\beta k_0 \approx \beta k_0^{\mathrm{ns}}$. If the level of $[\mathrm{Y_p}]$ is at its maximum level, it is impossible for the mutant cells to respond to repellents. Furthermore, since $[\mathrm{Y_p}]$ is constant as a function of $\beta k_0$ around the non-stimulated state, $\beta k_0$ must be lowered

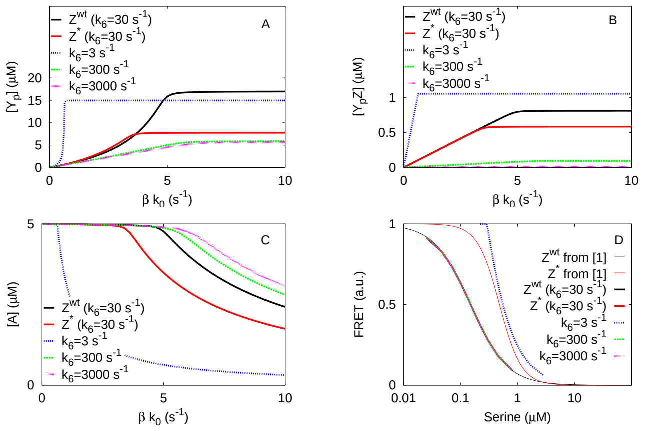


FIG. 7: Effect of changing $k_6$ on the response of the canonical chemotaxis model (Equations 1-3 of main text). Shown are: $[\mathrm{Y_p}](\beta k_0)$ (A), $[\mathrm{Y_pZ}](\beta k_0)$ (B), $[\mathrm{A}](\beta k_0)$ (C), FRET([serine]) (D). Please note that for a lower phosphatase activity in CheZ mutant cells ($k_6 = 3$ s$^{-1}$, blue line), the predicted FRET response agrees quite well with that measured experimentally (thin red line) [1]. The baseline parameters are given in Fig. 4.

by a large amount upon the addition of attractant before the mutant cells can respond. Chemotaxis thus seems impossible for the mutant cells. However, it is known that bacteria with a non-localizing phosphatase are able to chemotax towards attractants, although less efficiently than wild-type bacteria [6]. Hence, while a lower catalytic activity of diffusive CheZ with respect to localized CheZ can explain the experimentally observed change in the dose-response curve [1], it seems inconsistent with the observation that the mutants are still able to chemotax.

A similar behaviour is seen in Fig. 6 for a ten times lower value of the association rate $k_4$ of $\mathrm{CheY_p}$ to CheZ: although the dose response curves for wild-type and mutant bacteria can be simultaneously fitted, the mutant cells would adapt to the maximum values of both $\mathrm{CheY_p}$ and $\mathrm{CheY_pCheZ}$. Furthermore, $[\mathrm{Y_pZ}]$ is much lower for the mutant cell than for the wild-type cell as can be seen from Fig. 6B, in contrast with the observations in [1]. We therefore argue that the canonical model of the intracellular chemotaxis network needs to be refined.


[1] Vaknin A, Berg HC (2004) Single-cell FRET imaging of phosphatase activity in the *Escherichia coli* chemotaxis system. Proc Natl Acad Sci USA 101:17072–17077.

[2] Sourjik V, Berg HC (2002) Binding of the *Escherichia coli* response regulator CheY to its target measured in vivo by fluorescence resonance energy transfer. Proc Natl Acad Sci USA 99:12669 – 12674.

[3] Van Albada SB, Ten Wolde PR (2007) Enzyme localization can drastically affect signal amplfication in signal transduction pathways. PLoS Comp Biol 3:e195.

[4] Goldbeter A, Koshland, Jr DE (1981) An amplified sensitivity arising from covalent modification in biological systems. Proc Natl Acad Sci USA 78:6840–6844.

[5] Wang H, Matsumura P (1996) Characterization of the $\mathrm{CheA_s}$/CheZ complex: a specific interaction resulting in enhanced dephosphorylating activity on CheY-phosphate. Mol Microbiol 19:695–703.

[6] Sanatinia H, Kofoid EC, Morrison TB, S PJ (1995) The smaller of two overlapping CheA gene products is not essential for chemotaxis in *Escherichia coli.* J Bacteriol 177:2713–2720.
