# Supplementary material for: Differential Affinity and Catalytic Activity of CheZ in E. coli Chemotaxis
Source: PLoS Comput Biol. 2009 May 8;5(5):e1000378. doi: 10.1371/journal.pcbi.1000378 (PMC2673030; doi:10.1371/journal.pcbi.1000378)
Supplement: Text S3 — Cooperativity in the phosphatase reactions. (0.29 MB PDF) [file pcbi.1000378.s003.pdf]

Text S3
Differential affinity and catalytic activity of CheZ in E. coli *chemotaxis*

S.B. van Albada and P.R. ten Wolde
(Dated: February 9, 2009)

Cooperativity in the phosphatase reactions

Cooperativity in the dephosphorylation of $\mathrm{CheY_p}$ by CheZ was first shown by Blat et al. [1]. A quantitative study of the cooperativity in $\mathrm{CheY_p}$ dephosphorylation was presented in [2]. In this section we discuss a model of CheZ activity that can describe the experimental data of Blat et al. [2]. We will show that this model can accurately reproduce the experimental data of Eisenbach *et al.*, although, as we will discuss, it seems likely that some of the rate constants obtained might differ from those *in vivo*. We also note here for clarity that since the experiments were performed *in vitro* and no $\mathrm{CheA_s}$ was present, the results apply to dephosphorylation of $\mathrm{CheY_p}$ by diffusive CheZ and not to CheZ localized at the receptor cluster.

The model for the phosphatase activity is given by Eqs. 1 and 3 of the main text. Together with the phosphorylation reaction of CheY, this yields the following model for the experimental setup of Ref. [2]:

$$\mathrm{Y} \overset{\mathrm{AcP}}{\rightarrow} \mathrm{Y_p} \tag{1}$$

$$\mathrm{Y_p} + \mathrm{Z} \underset{k_5}{\overset{k_4}{\rightleftharpoons}} \mathrm{Y_pZ} \overset{k_6}{\rightarrow} \mathrm{Y} + \mathrm{Z} \tag{2}$$

$$\mathrm{Y_pZ} + \mathrm{Y_p} \underset{k_8}{\overset{k_7}{\rightleftharpoons}} (\mathrm{Y_p})_2\mathrm{Z} \overset{k_9}{\rightarrow} \mathrm{Y} + \mathrm{Y_pZ} \tag{3}$$

Here, the first reaction describes the phosphorylation reaction in the *in vitro* experimental setup of Eisenbach *et al.* [2], in which CheY is continuously phosphorylated by acetyl phosphate AcP. Please note that Z corresponds to one CheZ *dimer*.

Figure 1c in [2] shows the results on the kinetics of CheY dephosphorylation by CheZ. $\mathrm{CheY_p}$, in the presence of acetyl phosphate, was instantaneously mixed with a small amount of CheZ. The total concentration $[\mathrm{CheY}]_\mathrm{T}$ was 5 μM and the concentration of CheZ dimers was 0.1 μM–much lower than in a living cell. The rate of phosphorylation of CheY by AcP was also low, 0.207 s^{-1} [2]. Four relevant quantities can be extracted from the phosphorylation kinetics in Fig. 1c of [2], i) the time duration of the delay, ii) the value of $d[\mathrm{Y_p}]/dt$ after the transient, iii) the size (and presence) of the undershoot before the steady state is reached and iv) the steady state concentration of $\mathrm{CheY_p}$.

Figure 1 shows the effect of individually varying the parameters on the phosphatase kinetics. We assume that the two backward rates k_5 and k_8 are zero, since the dissocation rates are smaller than the catalytic rates [3]. The initial delay is determined by the time it takes for $[(\mathrm{Y_p})_2\mathrm{Z}]$ to reach its maximum level. In the limit that

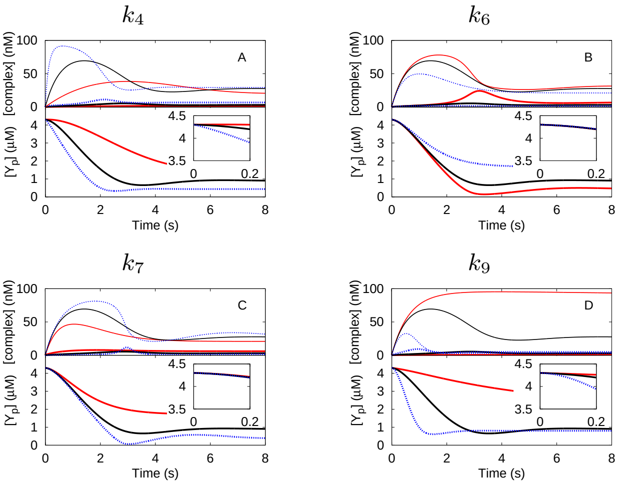

FIG. 1: Effect of varying the parameters of the cooperative model (Equations 2–3) on the phosphatase kinetics. The upper panels show the concentrations of $[(\mathrm{Y_p})_2\mathrm{Z}]$ (upper three curves) and of $[\mathrm{Y_pZ}]$ (lower three curves) as functions of time. The lower panels show the evolution of the concentration of $\mathrm{CheY_p}$ upon addition of CheZ. The concentration of $\mathrm{CheY_p}$ does not go to zero because CheY is continuously phosphorylated by AcP, as in the experimental setup [2]. The insets show a magnification of the initial delay. A. Effect of k_4. B. Effect of k_6. C. Effect of k_7. D. Effect of k_9. The parameter values are 0.2 (red), 1 (black) and 5 (blue) times the baseline parameters of Fig. 3.

the binding of $\mathrm{CheY_p}$ to $\mathrm{CheY_pCheZ}$ is much faster than the association of $\mathrm{CheY_p}$ to CheZ, i.e. if $k_7 \gg k_4$, the delay is dominated by k_4 (see Fig. 1A). The association rate k_4 is, however, sufficiently fast, such that after this transient, a steady state can be reached in which CheZ is predominantly in the state $(\mathrm{Y_p})_2\mathrm{Z}$. In this regime, the overall phosphatase activity per CheZ dimer is to a good approximation given by k_9, and the slope of $[\mathrm{Y_p}](t)$ is given by $k_9[\mathrm{Z}]_\mathrm{T}$.

The undershoot of $[\mathrm{Y_p}](t)$ in Fig. 1 arises from the subtle interplay between a number of factors. After a short transient of about 0.1 s, essentially all CheZ dimers are in the $(\mathrm{Y_p})_2\mathrm{Z}$ state. In this state, the phosphatase activity of the CheZ dimers is high, and the concentration of $\mathrm{CheY_p}$ drops rapidly. Importantly, when a $\mathrm{CheY_p}$ molecule in a $(\mathrm{Y_p})_2\mathrm{Z}$ complex is dephosphorylated, a $\mathrm{Y_pZ}$ complex is produced. When the concentration of $\mathrm{CheY_p}$ is high, this complex can immediately rebind another $\mathrm{CheY_p}$ molecule, from which another catalysis reaction can take place. However, after about $1-2\,\mathrm{s}$, the concentration of $\mathrm{CheY_p}$ has dropped so much that the rate

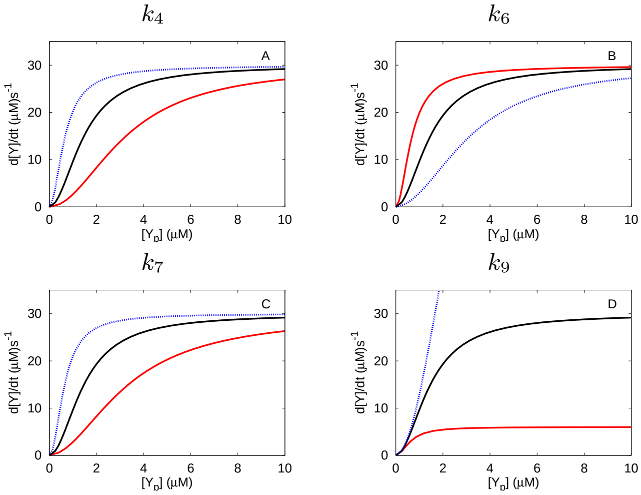

FIG. 2: Effect of varying the parameters of the cooperative model (Equations 2–3) on the specific phosphatase activity. A. Effect of k_4. B. Effect of k_6. C. Effect of k_7. D. Effect of k_9. The parameter values were 0.2 (red), 1 (black) and 5 (blue) times the baseline parameters of Fig. 3.

of association between CheY$_\mathrm{p}$ and CheY$_\mathrm{p}$CheZ decreases significantly. The concentration of $(\mathrm{Y_p})_2\mathrm{Z}$ now decreases, while the concentration of $\mathrm{Y_pZ}$ *increases*. Since the phosphatase activity of $\mathrm{Y_pZ}$ is lower than that of $(\mathrm{Y_p})_2\mathrm{Z}$, the concentration of CheY$_\mathrm{p}$ will now increase again, until a new steady state is finally reached.

One would expect that the steady state concentration of CheY$_\mathrm{p}$ can only decrease if any of the four association constants or catalytic activities is raised. Interestingly, this does not hold for k_6 (see Fig. 1B). While the total steady state catalytic activity of $\mathrm{Y_pZ}$, as given by $k_6[\mathrm{Y_pZ}]$, increases for larger values of k_6, the total catalytic activity of $(\mathrm{Y_p})_2\mathrm{Z}$, $k_9[(\mathrm{Y_p})_2\mathrm{Z}]$, decreases by a larger amount; the reason is that as k_6 increases, the concentration of $(\mathrm{Y_p})_2\mathrm{Z}$ decreases. Consequently, the total steady state catalytic activity of $\mathrm{Y_pZ}$ and $(\mathrm{Y_p})_2\mathrm{Z}$ together decreases as k_6 is raised. This increases the concentration of CheY$_\mathrm{p}$.

Figure 2a in [2] shows the specific phosphatase activity of CheZ as a function of the concentration of CheY$_\mathrm{p}$. The relevant quantities in Fig. 2a in [2] are the degree of cooperativity and the limiting value of the specific phosphatase activity. The effect of individually varying the parameters k_4, k_6, k_7 and k_9 on the specific phosphatase activity can be seen in Fig. 2. To analyse the dependence on these variables, we will first derive an expression for the phosphatase activity in steady state in the cooperative model given by Eqs. 2 and 3. First, $d[\mathrm{Z}]/dt = 0$ gives $[\mathrm{Y_p}][\mathrm{Z}] = (k_5 + k_6)/k_4[\mathrm{Y_pZ}] \equiv K_{M,1}[\mathrm{Y_pZ}]$. Next, $d[(\mathrm{Y_p})_2\mathrm{Z}]/dt = 0$ leads to $[\mathrm{Y_p}][\mathrm{Y_pZ}] = (k_8{+}k_9)/k_7\,[(\mathrm{Y_p})_2\mathrm{Z}] \equiv K_{M,2}[(\mathrm{Y_p})_2\mathrm{Z}]$. The total concentration of CheZ equals $[\mathrm{Z}]_\mathrm{T} = [\mathrm{Z}] + [\mathrm{Y_pZ}] + [(\mathrm{Y_p})_2\mathrm{Z}]$. Via elimination of $[(\mathrm{Y_p})_2\mathrm{Z}]$ and $[\mathrm{Z}]$, $[\mathrm{Y_pZ}]$ can be expressed

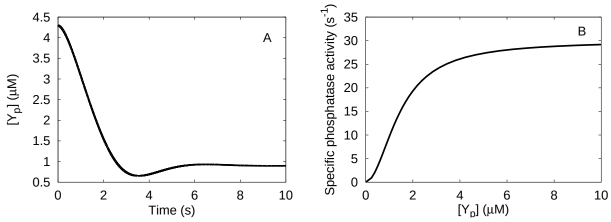

FIG. 3: Best fit of the cooperative model to Figs. 1c and 2a of [2]. The parameter values are: $k_4 = 0.36(2.0) \cdot 10^6\ \mathrm{M^{-1}s^{-1}}$, $k_6 = 7.5(5.0)\ \mathrm{s^{-1}}$, $k_7 = 3(3) \cdot 10^8\ \mathrm{M^{-1}s^{-1}}$, $k_9 = 30(10)\ \mathrm{s^{-1}}$.

as

$$[\mathrm{Y_pZ}] = \frac{[\mathrm{Z}]_\mathrm{T}[\mathrm{Y_p}]}{K_{M,1} + [\mathrm{Y_p}] + [\mathrm{Y_p}]^2/K_{M,2}}. \quad (4)$$

The concentration of the doubly-bound state is given by

$$[(\mathrm{Y_p})_2\mathrm{Z}] = [\mathrm{Y_pZ}][\mathrm{Y_p}]/K_{M,2}, \quad (5)$$

Since the production rate of CheY is equal to $k_3[\mathrm{Y_pZ}] + k_6[(\mathrm{Y_p})_2\mathrm{Z}]$, it follows that

$$\frac{d[\mathrm{Y}]}{dt} = \frac{[\mathrm{Z}]_\mathrm{T}[\mathrm{Y_p}](k_6 + k_9[\mathrm{Y_p}]/K_{M,2})}{K_{M,1} + [\mathrm{Y_p}] + [\mathrm{Y_p}]^2/K_{M,2}}. \quad (6)$$

The network behaves cooperatively if the numerator is quadratic in $[\mathrm{Y_p}]$ and the denominator only marginally depends on $[\mathrm{Y_p}]$ when $[\mathrm{Y_p}]$ is small. This is achieved if k_9 is much larger than k_6 and if $K_{M,1}$ is larger than $K_{\mathrm{M},2}$: in the limit that $k_6 \ll k_9$ and $K_{\mathrm{M},1} \gg K_{\mathrm{M},2}$, the dephosphorylation rate is given by

$$\frac{d[\mathrm{Y}]}{dt} = \frac{k_9[\mathrm{Z}]_\mathrm{T}[\mathrm{Y_p}]^2}{K_{\mathrm{M},1}K_{M,2} + [\mathrm{Y_p}]^2}. \quad (7)$$

This is a Hill function with a Hill coefficient of 2 and a concentration at which the rate is half maximal (the inflection point) given by $K_\mathrm{M}^\mathrm{eff} = \sqrt{K_{\mathrm{M},1}K_{\mathrm{M},2}}$. This can be verified from Fig. 2. An increase in either the association constant k_4 or k_7 by a factor C, or a decrease by the same factor of either the catalytic activity k_6 or k_9 leads to an increase in one of the Michaelis-Menten constants by the same factor and therefore to an increase by a factor $\sqrt{C}$ of $K_\mathrm{M}^\mathrm{eff}$. A change in k_9 by a factor C additionally leads to a C times higher limiting phosphatase activity. The value of k_9 can therefore be determined from the maximum phosphatase activity in Figure 2a in [2].

The best simultaneous fit to Figs. 1c and 2a of [2] is shown in Fig. 3. The used rate constants are $k_4 = 0.36(2.0) \cdot 10^6\ \mathrm{M^{-1}s^{-1}}$, $k_6 = 7.5(5.0)\ \mathrm{s^{-1}}$, $k_7 = 3(3) \cdot 10^8\ \mathrm{M^{-1}s^{-1}}$ and $k_9 = 30(10)\ \mathrm{s^{-1}}$. These values were also used as the baseline parameters in Fig. 2. While the best fits of the cooperative model show good agreement with the experimental data, it should be stressed that these experiments were performed *in vitro* rather than in a living bacterium. Therefore, it is

well possible that the actual rate parameters in a living cell differ from those determined here. For example, it is well known that the diffusion coefficient *in vitro* can be ten times higher than that in a living cell. This means that especially diffusion-limited association reactions can slow down *in vivo*. We assume that the value of k_7 is ten times lower *in vivo* than *in vitro*: *in vivo*, $k_7^{\rm cell} = 3 \cdot 10^7\ {\rm M}^{-1}{\rm s}^{-1}$; we assume that k_4 is unchanged, since that rate is not diffusion limited.

The full differential affinity and catalytic activity model for the intracellular chemotaxis pathway

The full model for the intracellular chemotaxis network of *E. coli* is given by the following chemical reactions (see the main text):

$$\mathrm{A} \overset{\beta k_0}{\rightarrow} \quad \mathrm{A_p} \tag{8}$$

$$\mathrm{A_p} + \mathrm{Y} \underset{k_2}{\overset{k_1}{\rightleftharpoons}} \quad \mathrm{A_pY} \quad \overset{k_3}{\rightarrow} \mathrm{A} + \mathrm{Y_p} \tag{9}$$

$$\mathrm{Y_p} + \mathrm{Z} \underset{k_5}{\overset{k_4}{\rightleftharpoons}} \quad \mathrm{Y_pZ} \quad \overset{k_6}{\rightarrow} \mathrm{Y} + \mathrm{Z} \tag{10}$$

$$\mathrm{Y_p} + \mathrm{Z_b} \underset{k_{11}}{\overset{k_{10}}{\rightleftharpoons}} \quad \mathrm{Y_pZ_b} \quad \overset{k_{12}}{\rightarrow} \mathrm{Y} + \mathrm{Z_b} \tag{11}$$

$$\mathrm{Y_pZ} + \mathrm{Y_p} \underset{k_8}{\overset{k_7}{\rightleftharpoons}} \quad \mathrm{(Y_p)_2Z} \quad \overset{k_9}{\rightarrow} \mathrm{Y} + \mathrm{Y_pZ} \tag{12}$$

$$\mathrm{Y_pZ_b} + \mathrm{Y_p} \underset{k_{14}}{\overset{k_{13}}{\rightleftharpoons}} \quad \mathrm{(Y_p)_2Z_b} \quad \overset{k_{15}}{\rightarrow} \mathrm{Y} + \mathrm{Y_pZ_b} \tag{13}$$

In this model, in wild-type cells CheZ is present both bound to the receptor cluster, indicated by $\mathrm{Z_b}$, and freely diffusive. The majority of CheZ is assumed to be diffusive, but the affinity for $\mathrm{CheY_p}$ as well as the catalytic activity is much larger for the localized fraction of CheZ (for parameter values, see the caption of Fig. 4).

Fig. 4 shows the response of the concentration of $\mathrm{CheY_p}$ and the FRET signal to changes in the activity of the receptor cluster, βk_0. We assume that the FRET signal is given by: $\mathrm{FRET} \propto [\mathrm{Y_pZ}] + 2[(\mathrm{Y_p})_2\mathrm{Z}] + [\mathrm{Y_pZ_b}] + 2[(\mathrm{Y_p})_2\mathrm{Z_b}]$. It is seen that the functions $\mathrm{FRET}(\beta k_0)$ and $[\mathrm{Y_p}](\beta k_0)$ consist of two parts. In the first regime, corresponding to low concentrations of $\mathrm{CheY_p}$, the fraction of CheZ that is localized to the receptor cluster is not yet saturated. In this regime, $\mathrm{CheY_p}$ that is produced by $\mathrm{CheA_p}$ at the cluster will rapidly bind to CheZ at the cluster. While the FRET signal increases in this regime due to binding of $\mathrm{CheY_p}$ to $\mathrm{CheZ_b}$, the concentration of $\mathrm{CheY_p}$ hardly increases due to the large phosphatase activity of $\mathrm{CheZ_b}$. The second part of the response curves corresponds to the regime in which $\mathrm{CheZ_b}$ is fully saturated. In this regime, a $\mathrm{CheY_p}$ molecule that is produced at the receptor cluster, can no longer bind a CheZ dimer that is bound to the receptor cluster; it will therefore diffuse into the cytoplasm, where it will bind a diffusive CheZ dimer. This CheZ has a lower phosphatase activity than $\mathrm{CheZ_b}$ and, as a result, the concentration of $\mathrm{CheY_p}$, as well as the FRET signal, will rise rapidly.

As discussed in the main text, if the activity $\beta k_0^{\rm ns}$ of the receptor cluster is the same for wild-type and CheZ mutant cells, it is difficult to have $[\mathrm{Y_p}]^{\rm ns}$ in the working range of the motor for both types of cells. As we discuss now, a cooperative (super-linear) dependence of the phosphatase activity on $[\mathrm{Y_p}]$ makes it easier to satisfy this constraint. First, we note that the constraint that $[\mathrm{Y_p}]$ should be within the working range of the motor for both wild-type and CheZ mutant cells is satisfied more easily if $[\mathrm{Y_p}]$ for diffusive CheZ is a concave (hyperbolic) function of βk_0. Such a concave functional form can be obtained when the phosphatase activity depends super-linearly on $[\mathrm{Y_p}]$: In steady state, the kinase activity $d[\mathrm{Y_p}]/dt$ is given by $\beta k_0[\mathrm{A}] = k_1[\mathrm{A_p}][\mathrm{Y}]$ and equal to the phosphatase activity $d[\mathrm{Y}]/dt$. If $[\mathrm{A}] \approx [\mathrm{A}]_{\rm T}$, the kinase activity is proportional to βk_0, which means that in steady state the specific phosphatase activity $d[\mathrm{Y}]/dt$ is proportional to βk_0. If the specific phosphatase acitivity (and hence βk_0) increases super-linearly (up to quadratically) with $[\mathrm{Y_p}]$ for low $[\mathrm{Y_p}]$ as in the cooperative CheZ model, $[\mathrm{Y_p}]$ as a function of βk_0 has a concave form, as can also be seen in Fig. 2.

We now briefly discuss the effect of varying the rate constants and the diffusion constant on the response curves $[\mathrm{Y_p}](\beta k_0)$ and $\mathrm{FRET}(\beta k_0)$, as shown in Fig. 4. The effect of changing the parameters related to the kinase reactions, k_1 and k_3, is similar to that of changing these rate constants in the canonical model, as discussed above. In particular, as long as $[\mathrm{A}] \approx [\mathrm{A}]_{\rm T}$, the total phosphorylation rate is independent of k_1 and k_3, and $[\mathrm{Y_p}]$ and FRET are fairly insensitive to changes in these rate constants.

The parameters k_4, k_6, k_7 and k_9 correspond to dephosphorylation reactions by CheZ in the cytoplasm. Clearly, changing these rate constants only affects the second regime of the response curves $[\mathrm{Y_p}](\beta k_0)$ and $\mathrm{FRET}(\beta k_0)$, in which CheZ bound to the cluster is saturated (see Fig. 4). The influence of varying these parameters on $[\mathrm{Y_p}](\beta k_0)$ can be deduced from the corresonding panels in Fig. 2, which shows the phosphatase activity for diffusive CheZ. Since the kinase activity $\beta k_0[\mathrm{A}]$ equals in steady state the phosphatase activity $d[\mathrm{Y}]/dt$, $[\mathrm{Y_p}](\beta k_0)$ is also given by $[\mathrm{Y_p}](d[\mathrm{Y}]/dt[\mathrm{A}]_{\rm T})$. Thus, by inverting the axes of Fig. 2, one can deduce the change in $[\mathrm{Y_p}](\beta k_0)$ upon varying k_4, k_6, k_7 and k_9. As expected, changing the catalytic rate k_9 has the largest effect on the response curve.

The parameters k_{10}, k_{12}, k_{13}, and k_{15} are rate constants associated with reactions of CheZ that is bound to the cluster; these rate constants correspond to, respectively, k_4, k_6, k_7 and k_9 of reactions of CheZ in the cytoplasm. As such, the effect of varying the parameters k_{10}, k_{12}, k_{13} and k_{15} on the response curve can be deduced from the effect of changing the parameters k_4, k_6, k_7 and k_9, discussed above. However, since CheZ bound to the

cluster is present in low concentrations, and has a much higher affinity for $CheY_p$ and a higher catalytic activity, the magnitude of the effect is markedly different. In particular, changing k_{10}, k_{12} and k_{13} hardly has any effect. This is because CheZ at the cluster is strongly driven to the $(CheY_p)_2CheZ$ state. For precisely the same reason, the largest effect is observed for changes in the catalytic rate k_{15}.

The last graph in Fig. 4 shows the effect of varying the diffusion coefficient D. The diffusion coefficient is assumed to be equal for all diffusive components. A decrease in D has the effect that larger gradients of $CheY_p$ arise. As a consequence, the concentration of $CheY_p$ integrated over the whole cell decreases. Since also larger gradients of Y_pZ and $(Y_p)_2Z$ occur, the total concentrations of Y_pZ and $(Y_p)_2Z$ decrease as well for lower values of the diffusion constant; this explains the decrease in FRET signal with decreasing diffusion constant.

---

[1] Blat Y, Eisenbach M (1996) Oligomerization of the phosphatase CheZ upon interaction with the phosphorylated form of CheY. J Biol Chem 271:1226–1231.

[2] Blat Y, Gillespie B, Bren A, Dahlquist FW, Eisenbach M (1998) Regulation of phosphatase activity in bacterial chemotaxis. J Mol Biol 284:1191–1199.

[3] Silversmith RE, Smith JG, Guanga GP, Les JT, Bourret RB (2001) Alteration of a nonconserved active site residue in the chemotaxis response regulator CheY affects phosphorylation and interaction with CheZ. J Biol Chem 276:18478–18484.

[4] Silversmith RE, Levin MD, Schilling E, Bourret RB (2008) Kinetic Characterization of Catalysis by the Chemotaxis Phosphatase CheZ. J Biol Chem 283:756–765.

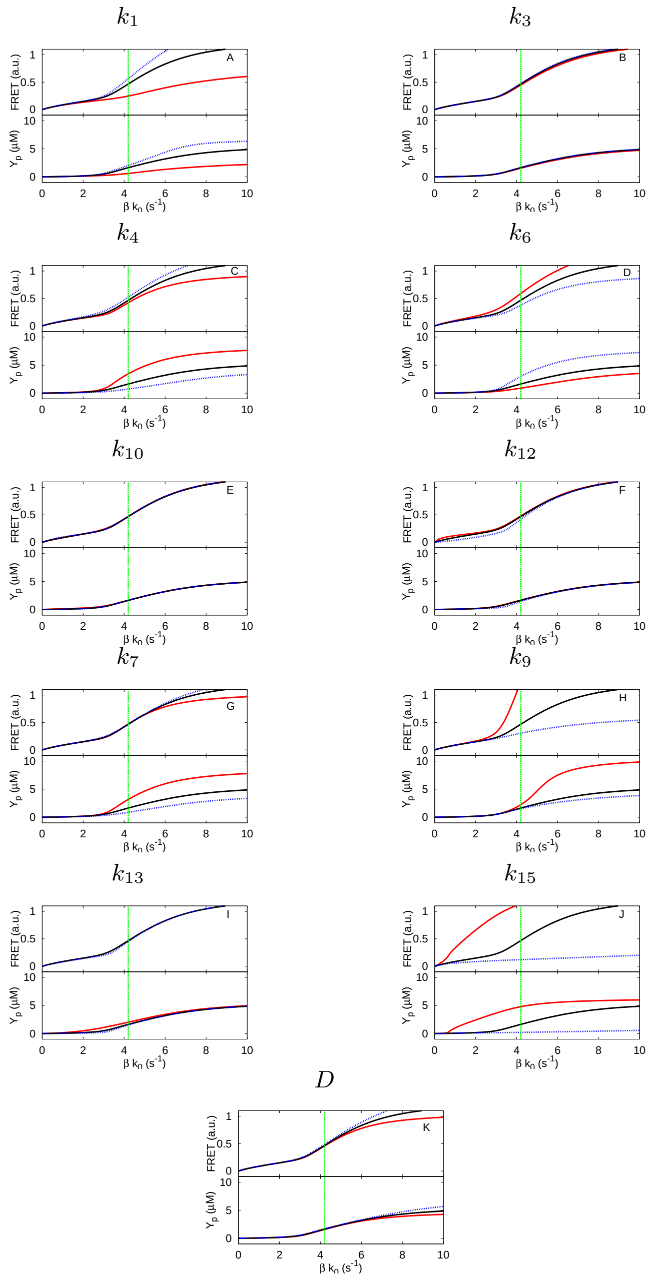

FIG. 4: The effect of varying the different parameters of the full model of the intracellular chemotaxis network (Equations 8–13) on the response curves $[\mathrm{Y_p}](\beta k_0)$ and $\mathrm{FRET}(\beta \mathrm{k}_0)$; here $\mathrm{FRET} \propto [\mathrm{Y_pZ}] + 2[(\mathrm{Y_p})_2\mathrm{Z}] + [\mathrm{Y_pZ_b}] + 2[(\mathrm{Y_p})_2\mathrm{Z_b}]$. The baseline parameters are $k_1 = 3\ 10^6\ \mathrm{M^{-1}s^{-1}}$, $k_3 = 750\ \mathrm{s^{-1}}$, $k_4 = 3.6\ 10^5\ \mathrm{M^{-1}s^{-1}}$, $k_6 = 7.5\ \mathrm{s^{-1}}$, $k_{10} = 6\ 10^8\ \mathrm{M^{-1}s^{-1}}$, $k_{12} = 40\ \mathrm{s^{-1}}$, $k_7 = 3\ 10^7\ \mathrm{M^{-1}s^{-1}}$, $k_9 = 30\ \mathrm{s^{-1}}$, $k_{13} = 9\ 10^8\ \mathrm{M^{-1}s^{-1}}$, $k_{15} = 160\ \mathrm{s^{-1}}$, $D = 5\ \mu\mathrm{m^2s^{-1}}$, $[\mathrm{Y}]_\mathrm{T} = 17.9\ \mu\mathrm{M}$, $[\mathrm{Z}]_\mathrm{T} = 1\ \mu\mathrm{M}$, $[\mathrm{Z_b}]_\mathrm{T} = 0.1\ \mu\mathrm{M}$ and $[\mathrm{A}]_\mathrm{T} = 5\ \mu\mathrm{M}$. The parameter values are 0.2 (red), 1 (black) and 5 (blue) times the baseline parameters. Exceptions are $k_{13} : k_{13} = 1.8\ 10^8,\ 6\ 10^9$ and $3\ 10^{10}\ \mathrm{M^{-1}s^{-1}}$ and the diffusion coefficient: $D = 3$ (red), 5 (black) and 10 $\mu\mathrm{m^2s^{-1}}$ (blue). The vertical green lines indicate the non-stimulated state for the baseline parameter set.
